# Transmission modes affect the population structure of potato virus Y in potato

**DOI:** 10.1371/journal.ppat.1008608

**Published:** 2020-06-23

**Authors:** Washington da Silva, Denis Kutnjak, Yi Xu, Yimin Xu, James Giovannoni, Santiago F. Elena, Stewart Gray

**Affiliations:** 1 Department of Pathology and Ecology, The Connecticut Agricultural Experiment Station, New Haven, Connecticut, United States of America; 2 School of Integrative Plant Science, Plant Pathology and Plant-Microbe Biology Section, Cornell University, Ithaca, New York, United States of America; 3 Department of Biotechnology and Systems Biology, National Institute of Biology, Ljubljana, Slovenia; 4 Instituto de Biología Integrativa de Sistemas (I^2^SysBio), CSIC-Universitat de València, Paterna, València, Spain; 5 Department of Plant Pathology, Nanjing Agricultural University, Nanjing, China; 6 Boyce Thompson Institute, Cornell University, Ithaca, New York, United States of America; 7 Emerging Pests & Pathogens Research Unit, USDA, ARS, Ithaca, New York, United States of America; 8 The Santa Fe Institute, Santa Fe, New Mexico, United States of America; Agriculture and Agri-Food Canada, CANADA

## Abstract

Transmission is a crucial part of a viral life cycle and transmission mode can have an important impact on virus biology. It was demonstrated that transmission mode can influence the virulence and evolution of a virus; however, few empirical data are available to describe the direct underlying changes in virus population structure dynamics within the host. Potato virus Y (PVY) is an RNA virus and one of the most damaging pathogens of potato. It comprises several genetically variable strains that are transmitted between plants via different transmission modes. To investigate how transmission modes affect the within-plant viral population structure, we have used a deep sequencing approach to examine the changes in the genetic structure of populations (in leaves and tubers) of three PVY strains after successive passages by horizontal (aphid and mechanical) and vertical (via tubers) transmission modes. Nucleotide diversities of viral populations were significantly influenced by transmission modes; lineages transmitted by aphids were the least diverse, whereas lineages transmitted by tubers were the most diverse. Differences in nucleotide diversities of viral populations between leaves and tubers were transmission mode-dependent, with higher diversities in tubers than in leaves for aphid and mechanically transmitted lineages. Furthermore, aphid and tuber transmissions were shown to impose stronger genetic bottlenecks than mechanical transmission. To better understand the structure of virus populations within the host, transmission mode, movement of the virus within the host, and the number of replication cycles after transmission event need to be considered. Collectively, our results suggest a significant impact of virus transmission modes on the within-plant diversity of virus populations and provide quantitative fundamental data for understanding how transmission can shape virus diversity in the natural ecosystems, where different transmission modes are expected to affect virus population structure and consequently its evolution.

## Author summary

Viruses can be transmitted between hosts via different modes, that can differently shape the population of transmitted viruses and thus have important implications for their biology and evolution. Potato virus Y, a damaging potato pathogen, can be transmitted between potato plants by aphid feeding, by wounding, or via tubers (mother to daughter plants). In this study, we have investigated how these three transmission modes directly affect the structure of the virus populations within the potato plants. We discovered that the mode of virus transmission significantly affected the population of the virus transmitted to the new plant. For example, virus populations transmitted by aphids were the least diverse while the ones transmitted vertically through tubers were the most diverse. To fully understand the effect of transmission modes on virus populations we needed to consider the events following the transmission, such as viral movement within a plant. Understanding the role that transmission modes play on the diversity and evolution of virus populations helps us to elucidate the mechanisms shaping the diversity of the virus in the field, and provides valuable information for future plant virus epidemiological studies that could promote the development of novel management strategies.

## Introduction

Transmission is a crucial step in virus survival and spread. It can define highly relevant characteristics of virus-host interactions such as virulence, virus fitness [1,2], and the strength of selection for resistance and tolerance genes in the host [3,4]. It also plays an important role in virus evolution and epidemiology [5]. Plant viruses can spread via different transmission modes, either horizontally (*e*.*g*., by insects, nematodes, direct contact, or risky agricultural practices), vertically (*e*.*g*., by seeds, pollen, tubers, or via vegetative propagation), or by mixed transmission modes [6]. Experimental studies addressing the effect of different transmission modes on the virulence of a plant virus demonstrated increased virulence for viruses transmitted horizontally in contrast to viruses transmitted vertically [2,7]. Moreover, several studies laid a foundation for understanding how virus populations in plants are affected by different transmission modes; *e*.*g*., by estimating very narrow population bottlenecks [8,9] and strong selection for insect transmission [10], narrow population bottlenecks for horizontal transmission by seeds [11] and strong population bottlenecks for contact transmission [12]. However, we are unaware of any study that systematically investigated the underlying population genetics changes for different transmission modes of the same virus.

Most of the known viruses infecting plants are RNA viruses. The high replication rate of RNA viruses coupled with a lack of proofreading ability by their RNA-dependent RNA-polymerases can lead to genetically diverse virus populations, *i*.*e*., mutant swarms or mutant clouds, within an infected host [13]. RNA virus populations within a host are often referred to as *quasispecies* [13], which are subjected to natural selection as diverse mutant swarms in their entirety [14], rather than as individual members of the population [13]. Two evolutionary processes, selection and genetic drift, are the major forces shaping virus population structures [15]. Selection is a directional process that results in an increase in the frequency of the fittest variants in the populations. Genetic drift is driven by random changes in populations’ composition and is mainly influenced by bottleneck events occurring during the virus life cycle, such as transmission between hosts and movement within the host [16]. It is difficult to distinguish the effects of selection and genetic drift in plant virus evolution studies since the same forces (*e*.*g*., vector transmission and within-host movement) have been linked to both evolutionary processes [15]. Nevertheless, technological challenges in measuring the complete genetic variation in a virus population have been lessened by the advent of high-throughput sequencing (HTS) technology, which allows detection of viral mutations at very low frequencies [17]. Combined with novel and evolving bioinformatics algorithms, HTS has improved our ability to study the processes that shape viral populations within hosts with an unprecedented level of genetic resolution [18–20].

Potato virus Y (PVY; genus *Potyvirus*, family *Potyviridae*) is a major virus pathogen of potatoes (*Solanum tuberosum* L. subsp. tuberosum) [21]. The virus exists as a complex of strains with PVY^O^ and PVY^N^ being parents to numerous emerging recombinant strains, several of which have become prevalent in potato fields worldwide [22]. PVY can be transmitted horizontally between plants by aphid vectors, mechanically by infected sap entering through wounds, and vertically through infected tubers. The PVY genome consists of a positive sense single-stranded RNA molecule with an open reading frame (ORF) encoding a large polyprotein subsequently cleaved into ten putative proteins [23], and another small ORF, named PIPO [24]. Numerous functional motifs along the potyvirus polyprotein have been identified and some are involved in the specific interactions of virus with its aphid vector during transmission [23]. The DAG motif located near the surface-exposed N-terminus of the coat protein was shown to interact with the PTK motif on the C-terminus of the helper component proteinase protein (HC-Pro). The KITC motif on the N-terminus of the HC-Pro interacts with binding domains on the aphid mouthparts allowing the HC-Pro to act as a bridge connecting the virus particle to the aphid and facilitating transmission [25].

In summary, PVY can be transmitted vertically and horizontally with known specific interactions between the virion and the aphid vector, and exhibits, as an RNA virus, the potential for rapid evolution. All these features make PVY an ideal system to test important research questions regarding the effect of different transmission modes on the structure of virus populations within its host. Here we describe experiments to characterize the changes in within-plant population structures of different PVY strains prior to and following passage of the virus by three different transmission modes (insect, vegetative propagation through tubers, and mechanical) to shed light on the role of transmission modes in the evolution and diversification of the PVY populations. We aimed to investigate: (1) if the structure of the PVY within-plant populations changed according to the transmission mode, (2) if the diversity of the viral populations differed among different organs (leaves *vs* tubers) for different transmission modes, (3) if the strength of the transmission bottlenecks occurring in each transmission mode can be inferred from population genetics data, and (4) if differences existed between different strains of PVY in regard to the effect of transmission mode on the virus population structure. We used Illumina HTS technology and state of-the-art population genetics algorithms to address these objectives.

## Material and methods

### Plants and PVY strains

Potato plants, cv Goldrush, vegetatively propagated from cuttings [26], were grown in four-gallon plastic pots containing Cornell soil mix [27], then inoculated at the 5–6 leaf stage. All plants were tested free from PVY infection by monoclonal double-antibody sandwich ELISA (Agdia, Elkhart, IN-USA) prior to inoculation. They showed no disease symptoms and no other viruses were expected to be present in the plants. They were maintained in an insect-free greenhouse under 16:8 h light:dark conditions at 25 ±3°C. Plants from each treatment and PVY strain were kept on individual benches (one bench per treatment per strain) in the greenhouse. The greenhouse was fumigated with insecticide (a mixture of pymetrozine (Endeavor 50WG) at 17.20 mg/m^2^ and bifenthrin (Talstar P) at 0.161 mL/m^2^) once a week to avoid aphid infestation and any cross-contamination among treatments and PVY strains. The three PVY isolates used in this study were collected from infected potato plants grown in Wisconsin (WI3, strain PVY^O^), Minnesota (MN21, strain PVY^N-Wi^), and Montana (MT100006, strain PVY^N^). The isolates were maintained long term in lyophilized tobacco tissue stored at −40°C and their identities were confirmed using a strain-specific RT-PCR multiplex diagnostic assay[28].

### Experimental design

**Fig 1:**
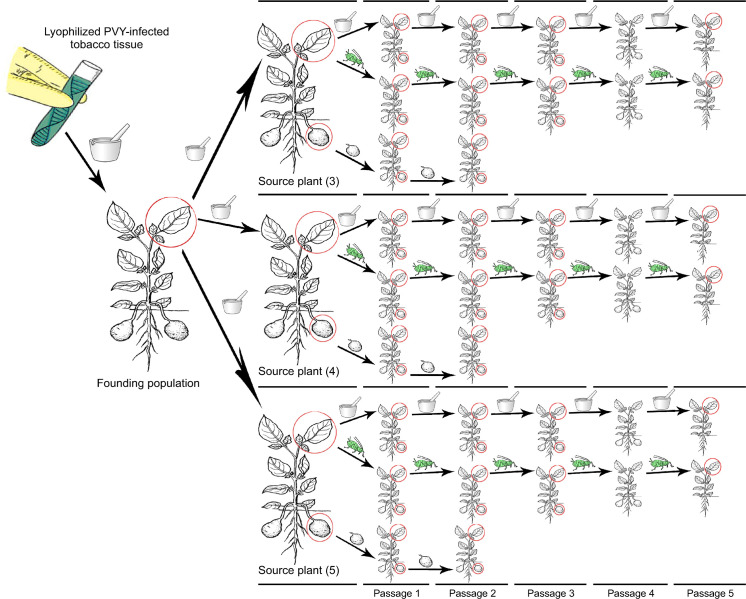
Experimental Design. PVY infected lyophilized tobacco tissue was used to mechanically inoculate a single founding potato plant. Virus from the founding plant was used to mechanically inoculate three potato plants (source plants, designated number 3, 4 and 5). Each source plant was considered a replication and used as virus source for each transmission mode: AT—aphid transmission (represented by the aphid cartoon), MI—mechanical inoculation (represented by mortar and pestle cartoon), and IT—infected tuber (represented by the potato tuber cartoon). Each time the virus was transmitted to a new plant it was counted as a passage. A leaf and a tuber (represented by the red circle) were sampled, at harvest, from designated plants to compare the virus populations in those locations and among each transmission mode over time. The same procedure was conducted for each of the three PVY strains, PVY^O^, PVY^N^_,_ and PVY^N-Wi^.

Lyophilized PVY-infected tobacco tissue (for each of three strains) was homogenized in 10 volumes of phosphate-buffered saline (PBS, pH 7.4) and used to mechanically inoculate a founding potato plant (Fig 1). Three weeks post-inoculation (wpi), each plant was tested by ELISA to determine their infection status. At harvest (14 weeks after planting), the youngest fully expanded leaf was sampled from each plant. Half of the terminal leaflet (~100 mg) was used for total RNA extraction, and the remainder of the leaflet was used to mechanically inoculate three potato seedlings, each considered a biological replication (Fig 1). These three plants (termed source plants, designated number 3, 4 and 5) were used as the virus source for the initial passages by three transmission modes: aphid transmission (AT), mechanical inoculation (MI), and infected tuber (IT).

The following procedures were used for all subsequent passages. At harvest (14 weeks after planting), the terminal leaflet of the youngest fully expanded leaf of each source plant was used as virus source tissue for the aphid transmission passage while the leaflet was attached to the plant. Adult apterous green peach aphids (*Myzus persicae* Sulzer), reared on turnip (*Brassica rapa* L.), were collected, starved for two hours, then allowed a five min acquisition access period on the terminal leaflet. Ten aphids were moved to a clip cage enclosing the terminal leaflet of the youngest fully mature leaf on each of the recipient plants and allowed a 24 h inoculation access period. Subsequently, aphids were killed by fogging plants with a mixture of pymetrozine (Endeavor 50WG) at 17.20 mg/m^2^ and bifenthrin (Talstar P) at 0.161 mL/m^2^, and plants moved to a greenhouse.

The terminal leaflet, used as the virus source for AT, was collected, half of the leaflet was frozen in liquid nitrogen and stored in −80 ^o^C until used for total RNA extraction, the other half of the leaflet was used as virus source tissue for the mechanical inoculation assay. Tissue was homogenized in 10 volumes of phosphate-buffered saline (PBS, pH 7.4) and used to mechanically inoculate the terminal leaflet of the youngest fully mature leaf of a potato seedling (one seedling for each source plant—Fig 1). Three wpi, mechanically and aphid inoculated plants were tested by TAS-ELISA to determine their infection status. The plants were grown to maturity (~14 weeks) and the terminal leaflet of the youngest fully mature leaf was used for the next aphid transmission and mechanical passage and total RNA extraction, in each respective transmission mode (Fig 1). At the same time, the plants were harvested and the largest infected tuber from each plant was sampled for RNA extraction.

To carry out the infected tuber transmission assay, the largest infected tuber from each source plant was harvested. The distal half of the tuber was cut longitudinally; one section was used for total RNA extraction and the other section as a seed piece for the next generation. The seed piece was soaked for one hour in 2 ppm gibberellic acid solution to break dormancy and planted in a four-gallon plastic pot containing Cornell soil mix. After four weeks, emerging sprouts were tested by TAS-ELISA to determine infection status and if positive, all but the most vigorous sprouts were removed and the plant was grown to maturity (~14 weeks). Then, the terminal leaflet from the youngest fully expanded leaf was collected for total RNA extraction, and tubers were harvested. The largest infected tuber from each plant was sampled and used for tuber transmission assay as described above (Fig 1). Five passages were performed using either mechanical or aphid transmission (Fig 1). Although five vertical passages of virus through tubers were planned, a lack of viable tuber production after the second generation limited the number of passages to two. Leaf samples were collected from the founder population plants and leaf and tuber samples were collected from all the source plants (Fig 1). For the AT and MI modes, leaf and tuber samples were collected from passages 1, 2, and 3. Only leaf samples were collected from passage 5 as no tubers were produced from the passage 5 plants (Fig 1). For the IT transmission mode, leaf and tuber samples were collected for passage 1 and 2 (Fig 1). All of these collected samples were used for the HTS-based analyses.

### RNA extraction and viral genome amplification

Total RNA was extracted from frozen leaf and tuber tissue using the PureLink Plant RNA Reagent (Thermo Fisher Scientific, Waltham, MA), following manufacturer’s directions. Total RNA concentration and quality was checked on a NanoDrop 200 Spectrophotometer (Thermo Fisher Scientific, Waltham, MA) and by agarose gel electrophoresis prior to RT-PCR amplification. We designed a set of PVY-specific primers for each strain that allowed amplification of fragments (4 for PVY^N^ strain and 5 for PVY^O^ and PVY^N-Wi^ strains, 1122 to 3775 bp long) along the entire genome with at least a 76 base pair overlap between amplicons—each primer set amplifies only its specific PVY strain (S1 Table). First-strand cDNA was synthesized from the extracted RNA using a mix of random hexamers and anchored-dT primers following the protocol provided by the supplier using a ProtoScript II First Strand cDNA Synthesis Kit (NEB, Ipswich, MA). cDNA was amplified (20 cycles) using Phusion Green Hot Start II High-Fidelity PCR Master Mix (Thermo Fisher Scientific, Waltham, MA), following manufacturer’s protocol and using the specifically designed PVY primer sets for each strain (S1 Table). Amplicons from each tissue sample were pooled, cleaned using ChargeSwitch PCR Clean-Up Kit (Thermo Fisher Scientific, Waltham, MA), and quantified using a NanoDrop 200 Spectrophotometer. Amplicon sizes were checked on 1% agarose gel electrophoresis.

### Library construction and sequencing

Amplicons were sheared by sonication using a Covaris S2 Focused-ultrasonicator (Covaris, Woburn, MA), 130 μL microTUBE AFA Fiber Pre-Slit Snap-Cap 6×16 mm (cat# 520045), and the microTUBE Holder (cat# 500114). A 50 μL sample was sheared using the following parameters: Intensity of 5, duty cycle of 10%, 200 cycles per burst, temperature of 7 ^o^C, and treatment time of 3 min. DNA fragment length distribution was assessed using a 2100 Bioanalyzer (Agilent, Santa Clara, CA) with a target peak size ranging from 150 to 500 bp. Libraries were constructed from 1–2 μg of DNA following a published protocol [29], with modifications. Fragmented DNA was purified and size selected using AMPure XP beads (NEB, Ipswich, MA) before end-repair, dA-tailing, and the universal TruSeq adapter ligation step. Size selection was done again using AMPure XP beads to filter out extra adapters and small sized fragments. Finally, each library was PCR enriched 6–8 cycles with six-base single indexed primers. The barcoded libraries were pooled in equal molarity and quality checked using a 2100 Bioanalyzer, and sequenced on an Illumina HiSeq 2000/2500 system with the high-output mode; generating 100-bp single-end reads, at the Center for Advanced Technology, Institute of Biotechnology, Cornell University, Ithaca, NY.

### HTS data pre-processing and consensus genomes reconstruction

Raw Illumina reads were first demultiplexed using Illumina’s CASAVA pipeline v1.8.2, and then imported into CLC Genomics Workbench v11.0 (https://www.qiagenbioinformatics.com/), where most of the following analyses were conducted. We first trimmed adaptor and amplification primer sequences from the reads and then performed quality-based trimming of the reads ends (Quality limit = 0.01, Ambiguous limit = 0, and Minimum number of nucleotides in reads = 50). Next, using the FASTX-toolkit (http://hannonlab.cshl.edu/fastx_toolkit/) we discarded all of the reads containing quality values lower than 20. In CLC Genomics Workbench, we then constructed consensus sequences of the three viral strains (PVY^O^, PVY^N^, and PVY^N-Wi^) present in the founding populations by mapping the reads (Match score = 1, Mismatch cost = 2, Cost of insertions and deletions = Linear gap cost, Insertion cost = 3, Deletion cost = 3, Length fraction = 0.95, and Similarity fraction = 0.95) to respective most similar reference sequences available in NCBI GenBank (KY847982 for strain PVY^N-Wi^, KY847986 for strain PVY^N^, and KY848031 for strain PVY^O^). Consensus sequences of the three founding populations were used as references in all the subsequent analyses.

### SNP calling and filtering

Trimmed and quality filtered reads of each of the samples from the experiment were first mapped to consensus reference sequences of all three PVY strains used in the experiment (Match score = 1, Mismatch cost = 2, Cost of insertions and deletions = Linear gap cost, Insertion cost = 3, Deletion cost = 3, Length fraction = 0.99, and Similarity fraction = 0.97). To avoid possible contaminant reads of other strains, resulting from, *e*.*g*., barcode misassignments, all the reads mapping to other strains than the one present in the sample, were removed in this step. Such cleaned read datasets were then mapped to the consensus reference sequence of the founding population of the corresponding strain (Match score = 1, Mismatch cost = 2, Cost of insertions and deletions = Linear gap cost, Insertion cost = 3, Deletion cost = 3, Length fraction = 0.99, and Similarity fraction = 0.97) and CLC Genomics Low Frequency Variant Detection v2.02 tool was used to call the SNPs in each of the samples (Required significance (%) = 1.0, Minimum frequency (%) = 0.1, Ignore non-specific matches = Reads, Minimum coverage = 10, and Minimum count = 2).

To remove possible reverse-transcription, amplification, and sequencing artifacts we further filtered SNPs; only the SNPs that appeared in at least one time point (within a single lineage) at a frequency higher than 1% were retained for further analyses. Moreover, SNPs with the forward/reverse balance < 0.1% in majority of sequenced time points, SNPs positioned at the known polymerase slippage site in the PVY genome and SNPs at the sites with sequencing coverage < 100×, were removed from further analysis. Such cleaned SNP tables were then used as an input for subsequent analyses.

### Indices of viral population diversity and divergence

Filtered SNP tables were used as an input for SNPGenie [30] to calculate the nucleotide diversities, that represent a mean number of pairwise differences per site in a population of sequences. Nucleotide diversity was calculated for all sites (*π*), for synonymous sites (*π*_*S*_), and for nonsynonymous sites (*π*_*N*_). SNPGenie also calculated the mean number of nonsynonymous differences from the reference per nonsynonymous site (*d*_*N*_) and the mean number of synonymous differences from the reference per synonymous site (*d*_*S*_). The indices were averaged over a complete viral genome or over separate PVY cistrons. Nucleotide diversities (*π*) for leaf samples were plotted through time as line plots for each lineage and the average was calculated and plotted through time for all of the lineages corresponding to the respective transmission modes. Likewise, *d*_*N*_ and *d*_*S*_ were plotted through time. Generalized linear mixed model (GLMM) analysis in SPSS Statistics version 26 (IBM Corp., Armonk, NY) was used to test for factors contributing to differences in nucleotide diversities for the samples of passages 1–5. The three orthogonal factors included in the model were transmission mode (*T*), viral strain (*S*), and evolutionary passage (*P*) and all their interactions, using the following model equation: *π*_*ijkl*_ = Π + *T*_*k*_ + *S*_*i*_ + *P*_*j*_ + (*T*×*S*)_*ik*_ + (*T*×*P*)_*j*k_ + (*S*×*P*)_*ij*_ + (*T*×*S*×*P*)_*ijk*_ + *ε*_*ijkl*_, where *Π* represents the grand mean nucleotide diversity and *ε*_*ijkl*_ the error term assumed to be normally distributed. Nucleotide diversities were compared between leaf and tuber samples of the same plant by plotting the values for *π* in leaves against the value of *π* in tubers. We calculated the ratios of *π* in tubers to *π* in leaves and plotted their decimal logarithmic values for separate transmission modes as histograms. One-sample Wilcoxon signed rank tests were used to test if the median of the distributions of the ratios for each transmission mode is higher than one (greater diversity in tubers) or lower than one (greater diversity in leaves).

Nucleotide diversities (*π*_*S*_ and *π*_*N*_) were calculated separately for each of the PVY cistrons in each of the samples. The results were plotted for each cistron in time as line plots. To test for possible difference between the values for different cistrons, the cumulative distributions were calculated (including all of the lineages and all of the time points) and plotted for each cistron for both *π*_*S*_ and *π*_*N*_. Pairwise Kolmogorov-Smirnov tests were used to compare the cumulative distributions for all of the pairs of PVY cistrons for *π*_*S*_ and *π*_*N*_ values.

### Effective population sizes and SNPs selection coefficients

To estimate effective population sizes, *N*_*e*_, (reflecting population bottlenecks in different transmission modes) and locus-specific selection coefficients, *s*, of the observed SNPs, we employed the time-series information about frequency of observed SNPs. Filtered SNP tables of each lineage were converted to input files for ApproxWF analysis [31], including only the lineages with data for at least three time points and excluding the SNPs that only appeared in the last time point. *N*_*e*_ and *s* were estimated using task = estimate setting with h = 0.5 (dominance coefficient, since viruses are haploid), a log-uniform prior for *N*_*e*_, log*N*_*e*_ ~ *U*(1, 5), and normal prior for *s*, *s* ~ *N*(0, 0.1), and run with 51 states for 100,000 MCMC iterations. Results were further analyzed in R v3.6.1 [32]. First 10,000 MCMC simulations were treated as a burn-in and discarded. Posterior distributions for *N*_*e*_ of each lineage were plotted as kernel density plots and their modes were calculated. Modes for each of the lineages were plotted and used to compare the *N*_*e*_ between different transmission modes using paired two sample Wilcoxon signed-rank test. For *s*, mean, median, and 95% highest posterior density intervals were calculated and plotted for each analyzed SNP of all analyzed lineages.

### Visualization and clustering of SNPs trajectories

To better understand the dynamic changes in viral population structures we plotted the changes in frequency of each SNP through the experiment for each of the lineages using R v3.6.1. Each SNP was treated as a *n*-dimensional vector, dimensions representing frequency of the SNP at each sequenced time point. Heatmap.2 library [33] was used to cluster SNPs frequency trajectories and visualize them as a heat map for each lineage. To further search for possible positively selected SNPs we filtered out SNPs that appeared at frequency of at least 10% in at least two separate lineages, and then plotted dynamics of such SNPs frequencies as heatmaps.

## Results

**Fig 2:**
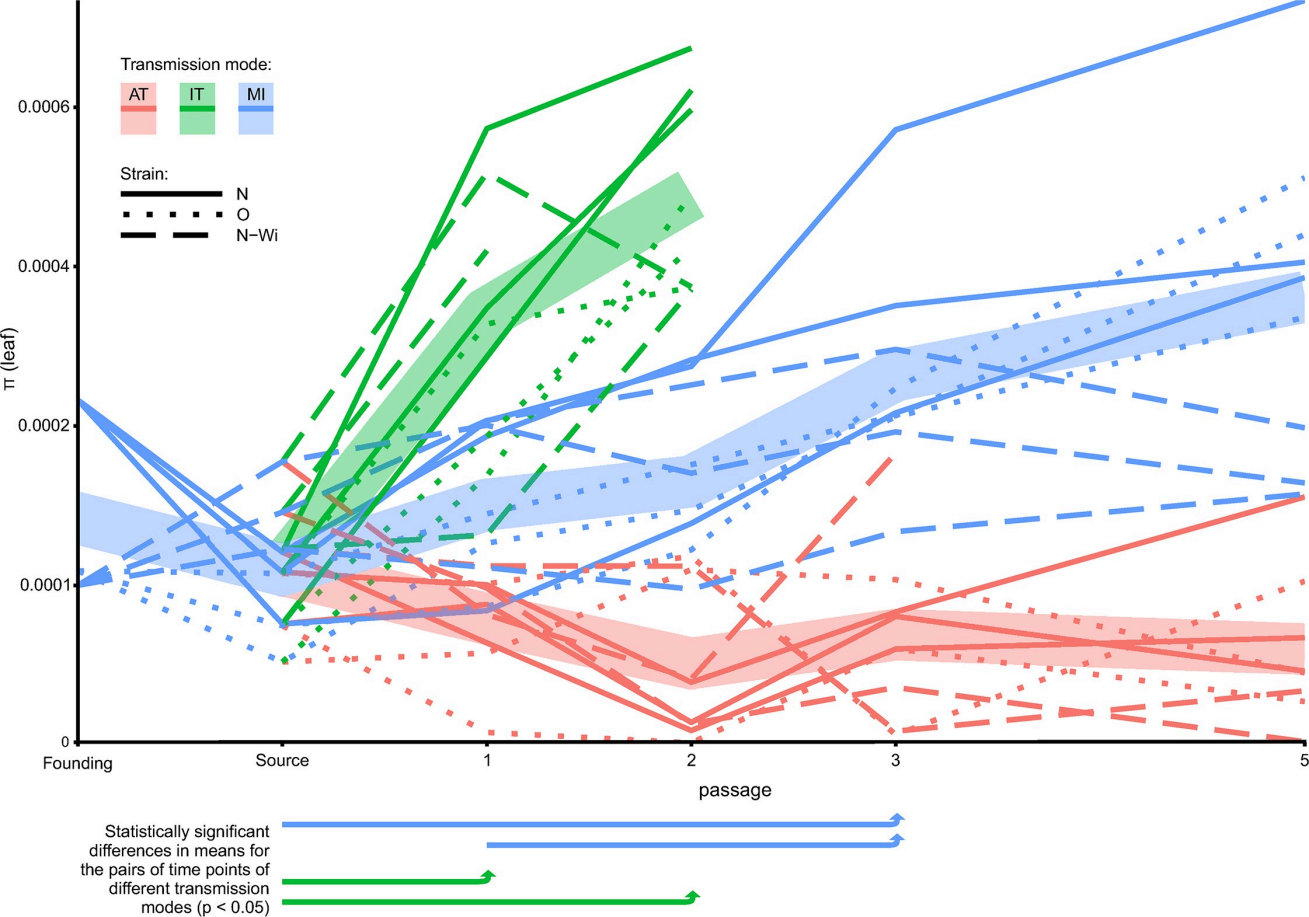
Temporal changes in PVY nucleotide diversities in leaves during experimental evolution using three different transmission modes. Thin lines connect PVY nucleotide diversities (*π*) in leaves for separate lineages over the course of the experiment. Colors represent the three different transmission modes (AT–aphid transmission, IT–transmission through infected tubers and MI–mechanical transmission). Line types represent the three different PVY strains used in the experiment. Thick translucent lines connect average values for all of the lineages within the same transmission mode over the course of the experiment. Below the plot, the arrows connect time points with significant increase in nucleotide diversity for specific transmission modes (based on Bonferroni-corrected *post hoc* pairwise comparisons of the estimated marginal means). Only the data obtained by the analysis of leaf samples is presented in this figure.

We collected and sequenced 183 samples from potato leaves and tubers during the course of the experiment (61 samples per PVY strain, as shown in Fig 1). After the quality control of the data and filtering, we obtained 59 samples for PVY^O^, 58 samples for PVY^N^, and 57 samples for PVY^N-Wi^. Detailed information about each of the analyzed samples is given in S2 Table. The mapped read depths across the PVY genomes for all the samples are shown in S1–S3 Figs. SNP tables for each of the samples are available as S1 File. Raw sequencing reads are deposited in NCBI SRA database, linked to the NCBI BioProject accession PRJNA601749. The consensus genomic sequences for PVY founding populations for all three strains used in the experiment were deposited in NCBI GenBank (MT350288, MT350289, and MT350290). The founding PVY populations for all three strains contained only the respective isolate sequences and had slightly different, but relatively low genetic diversity (Fig 2).

### Nucleotide diversities of the PVY populations are significantly influenced by transmission modes

We first investigated the dynamics of the viral populations' diversities for the three different strains for different transmission modes. We observed higher diversities of PVY populations in lineages transmitted by the MI and IT modes, and less diverse populations in the AT lineages. In most of the AT lineages, the nucleotide diversities dropped after the first passage and remained relatively low during the course of the experiment (Fig 2). In contrast, the nucleotide diversities for the viral populations transmitted mechanically or by tubers, increased during the experiment (Fig 2). GLMM analysis suggested a significant effect of the transmission mode (*T*), passage number (*P*), and their interaction on the diversity of viral populations (Table 1). We did not detect significant effects of the strain (*S*) on the diversity of viral populations (Table 1). *Post hoc* pairwise Bonferroni-corrected comparisons of estimated marginal means showed significant differences between all of the pairs of the tested transmission modes (S3 Table), with AT lineages being the least diverse and IT lineages being the most diverse (Fig 2, S3 Table). *Post hoc* pairwise comparisons of means for different passages within the three transmission modes indicated significant increases in nucleotide diversities during the experiment for MI and IT lineages (Fig 2, S3 Table).

**Table 1:** Analysis of factors using GLMM analyses for the PVY nucleotide diversity, *π*, data (leaves).

Source	Likelihood ratio test *χ*^2^	*d*.*f*.	*p*
*Π*	177.901	1	< 0.001
*T*	95.561	2	< 0.000
*S*	4.138	2	0.126
*P*	9.433	3	0.025
*T × S*	7.568	4	0.109
*T × P*	16.805	4	0.002
*S × P*	9.433	6	0.151
*T × S × P*	6.185	7	0.518

*Π*, grand mean nucleotide diversity; *T*, transmission mode; *S*, virus strain; *P*, evolutionary passage; *d*.*f*., degrees of freedom.

### Differences in nucleotide diversities between leaves and tubers are transmission mode-dependent

**Fig 3:**
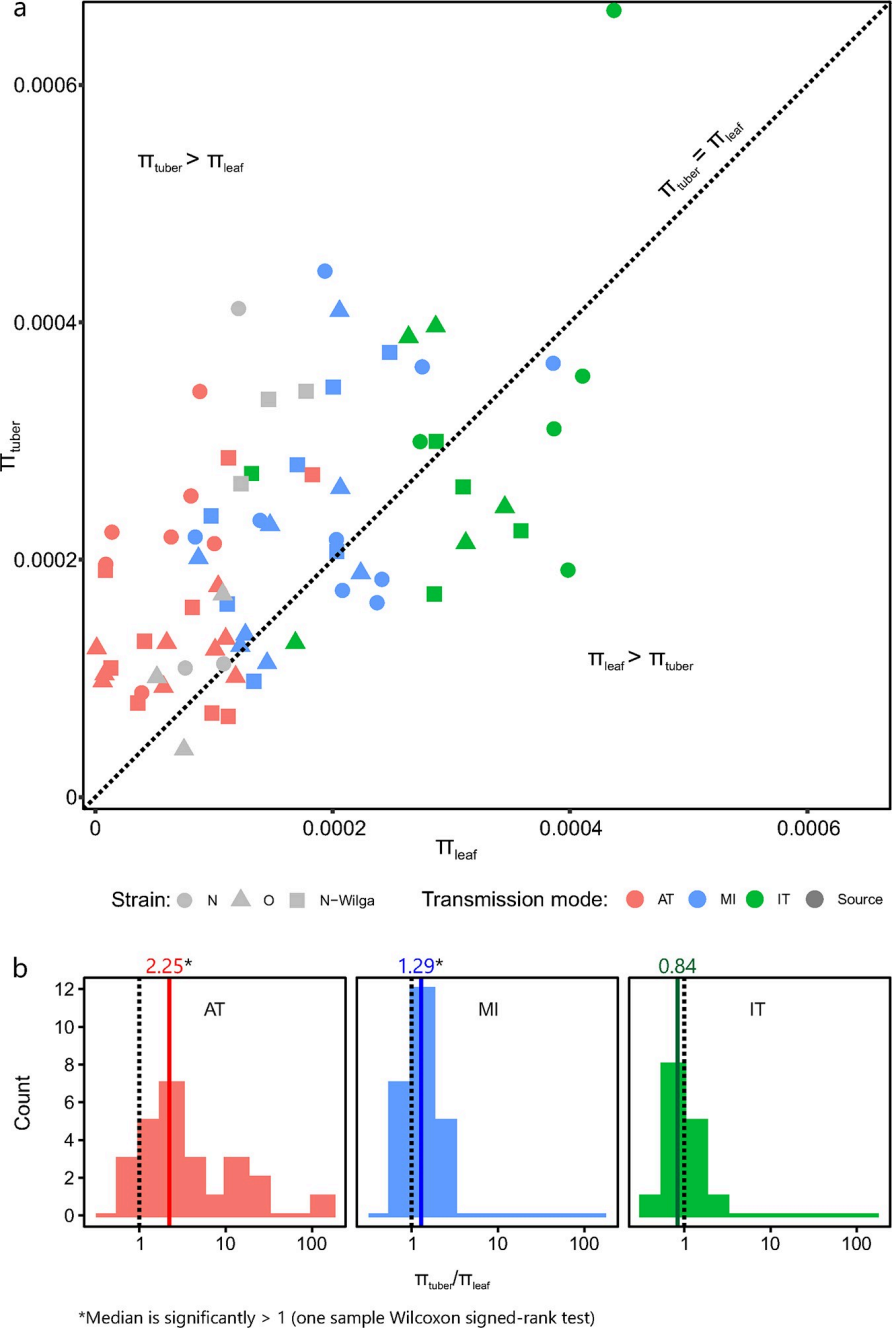
Comparison of nucleotide diversities (*π*) in leaves and tubers. (a) *π* in leaves (abscissae) are plotted against *π* in tubers (ordinates) for the same plants. Different colors represent different transmission modes, different symbols represent different strains. (b) Histograms show distributions of decimal log-transformed ratios of nucleotide diversities in tubers and leaves; dotted lines designate the null hypothesis of equal diversities *π*_*tuber*_/*π*_*leaf*_ = 1, colored lines represent medians of the separate distributions. Colors correspond to the different transmission modes as given in the legend above the histograms.

Diversities of viral populations in leaves of all source plants were very similar (Fig 2, Fig 3A: grey points). Diversities of viral populations in tubers of the source plants were very similar to those in leaves for most of the PVY^O^ and PVY^N^ strain source plants and slightly higher for PVY^N-Wi^ strain source plants (Fig 3A). Viral populations were more diverse in tubers than in leaves for the MI and AT virus populations. In contrast, diversities of the IT populations were mostly higher in leaves than in tubers of the same plant (Fig 3A). One-sample Wilcoxon signed ranked tests comparing the median of the distribution of *π*_*tuber*_/*π*_*leaf*_ to the null hypothesis of equal diversities (*π*_*tuber*_ = *π*_*leaf*_), for each transmission mode, supported significantly higher diversity of the viral populations in tubers than in leaves for MI and AT transmission modes. The median of the *π*_*tuber*_/*π*_leaf_ for IT was < 1, suggesting a lower diversity of the viral populations in tubers in this transmission mode; however, the difference was not statistically significant (Fig 3B). No grouping was observed for *π*_*tuber*_/*π*_leaf_ according to the virus strain (Fig 3A).

### Aphid and tuber transmission impose stronger genetic bottlenecks than mechanical transmission

**Fig 4:**
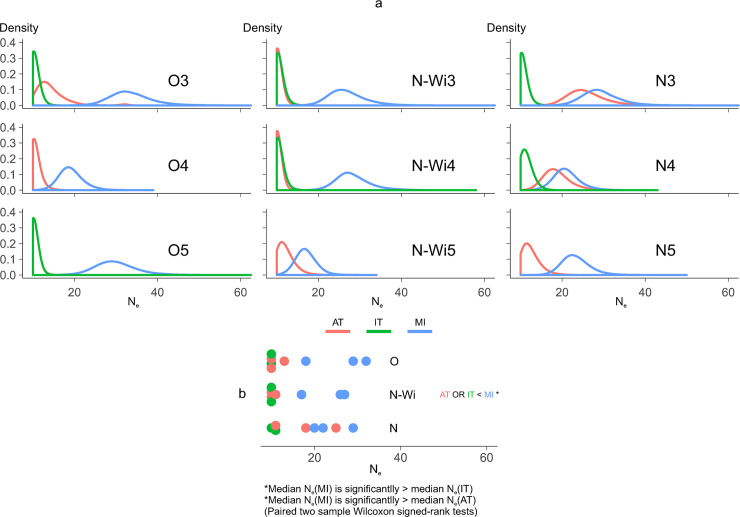
Estimations of effective population sizes (*N*_*e*_) for different lineages. (a) Kernel density plots (posterior distributions) for the estimation of the *N*_*e*_ values for each of the lineages in the experiment; lineages originated from the same source plant (designated number 3, 4 and 5) are plotted on the same plot, line colors represent transmission modes. (b) Modes of the *N*_*e*_ posterior distributions with colors corresponding to transmission modes. Statistically significant differences between medians of modes of *N*_*e*_ posterior distributions of the three transmission modes are designated on the plot.

We used time-series data of SNPs' frequencies changes to estimate effective population sizes for each of the lineages in the experiment using ApproxWF software [31]. This method provides an estimation of the demographic parameters of the population (simultaneously for *N*_*e*_ and locus-specific *s*) by using the information on how SNP frequencies are changing through time (passages). *N*_*e*_ represents the number of individuals from one passage that contribute to the establishment of the population in the next passage. This number is influenced by the sizes of genetic bottlenecks and can, in these experiments, be understood as a number of viral genome units that were transmitted from one to the other passage. We compared the viral populations in systemically infected leaves of source and inoculated plants, thus estimating *N*_*e*_ values for the whole transmission process (including transmission event itself, as well as the movement of the virus from the inoculated leaf to the systemically infected leaf). We observed that PVY was subjected to narrower population bottlenecks in the AT and IT modes, compared to MI mode. Estimations for most of the lineages of the AT and IT modes were very low, *i*.*e*., mostly 10 or less viral genome units (since 10 was the lower limit of the prior distribution for *N*_*e*_ in these analyses), pointing to very strong genetic bottlenecks occurring when PVY was transmitted by aphids or through tubers (Fig 4). *N*_*e*_ estimations for the MI mode were higher than for the AT and IT modes (Fig 4). Paired two-sample Wilcoxon signed rank tests showed that median of *N*_*e*_ values estimated for all MI lineages was significantly higher than for the AT and IT lineages (Fig 4). The trend of higher *N*_*e*_ values in the MI than the AT and IT modes was similar for all three strains tested in this study (Fig 4B). Estimations of *N*_*e*_ for the IT mode were very low (from lower than 10 to 11) for all three strains. For the AT mode, higher estimates of *N*_*e*_ were calculated for two out of the three experimental lineages of the PVY^N^ strain (18 and 25) (Fig 4). This implies a less narrow population bottleneck for aphid transmission of the PVY^N^ strain, relative to the PVY^O^ or PVY^N-Wi^ strains.

**Fig 5:**
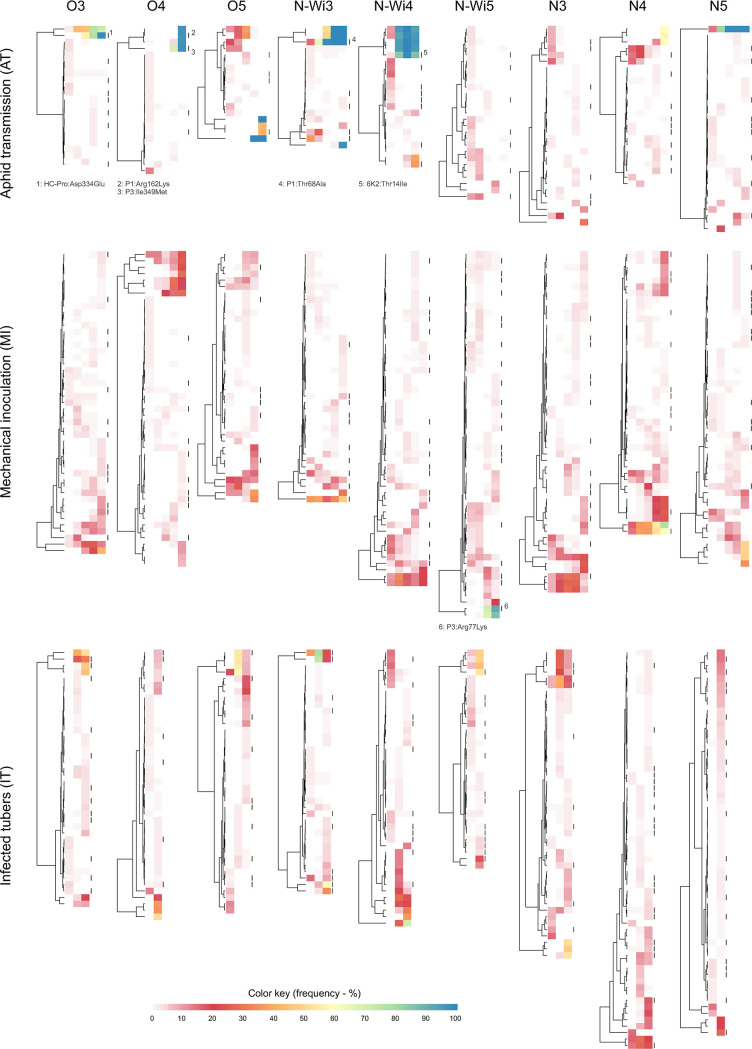
SNPs dynamics in different lineages. Each heatmap represents the dynamics of SNPs frequencies in each lineage during the experiment. Within each heatmap, lines represent specific SNPs; rows represent passage numbers. SNPs frequencies are represented by color according to the color key at the bottom of the figure. The dendrograms in front of the heatmaps represent clustering of SNPs trajectories (SNPs that have similar frequency changes in time and thus may be linked are clustered together). Black rectangles at the end of each heatmap designate nonsynonymous mutations; positions and corresponding changes for fixed nonsynonymous mutations are given below the corresponding heatmap. Lineages started from the same source plant are grouped vertically (designated by strain name: PVY^O^, PVY^N-Wi^ or PVY^N^, plus lineage designation within the strain: 3, 4 or 5). Lineages transmitted by the same transmission mode are grouped vertically (AT, MI, IT).

To gain further insight into the dynamics of the viral populations transmitted by different transmission modes, we visualized the changes in SNPs' frequencies in each of the experimental lineages as heatmaps (Fig 5). This kind of visual representation intuitively confirmed some of the previously described quantitative results, such as rapid decreases in diversity of the viral populations in the AT mode accompanied by the small number of total observed SNPs and higher diversity and greater number of total observed SNPs in the MI and IT modes. Moreover, we also observed that the patterns of viral population dynamics differed between the MI and IT modes. Whereas many of the SNPs in the MI mode were retained over several passages or the duration of the experiment, most of the SNPs in the IT mode were generated de novo after each passage and only a few of them were retained over several passages (Fig 5). This observation agrees with the lower quantitative estimation of the *N*_*e*_ in the IT lineages compared to the MI lineages (in the IT lineages fewer individuals from previous passage contribute to the virus population in the following passage).

### Natural selection could not be distinguished from genetic drift

Using ApproxWF we also obtained estimations of separate SNPs *s* values, which indicate if specific SNPs in the population are subjected to positive or negative selection. All of the estimations had wide intervals and we were not able to reliably detect any sites under selection (all of the analyzed SNPs 95% highest posterior density intervals contained zero, S4–S6 Figs). The wide intervals for *s* observed in this analysis might be a consequence of relatively low number of analyzed time points and very low estimated *N*_*e*_ values. The latter makes distinction between natural selection and genetic drift very difficult [31].

Consensus sequences of viral populations changed (new or minor alleles reached frequencies higher than 50% in population) during the course of the experiment in two of the nine MI lineages (2 consensus level SNPs per lineage), seven of nine IT lineages (1–4 consensus level SNPs per lineage) and seven of nine AT lineages (1–5 consensus SNPs per lineage). Their dynamics can be observed in detail on Fig 5 as yellow, green and blue coded SNPs. We observed more fixed minor or new alleles in the AT mode (17) than in the MI (1) or IT (0) modes (Fig 5). Some of the mutations fixed were nonsynonymous changes (5 out of 17 in the AT mode and 1 out of 1 in the MI mode). It was not possible to infer if the observed fixations were only the result of a strong genetic drift or if some of them arose due to the natural selection. Furthermore, several SNPs that reached at least 10% in more than one lineage of the experiment (S7 Fig) may represent SNPs under positive selection.

**Fig 6:**
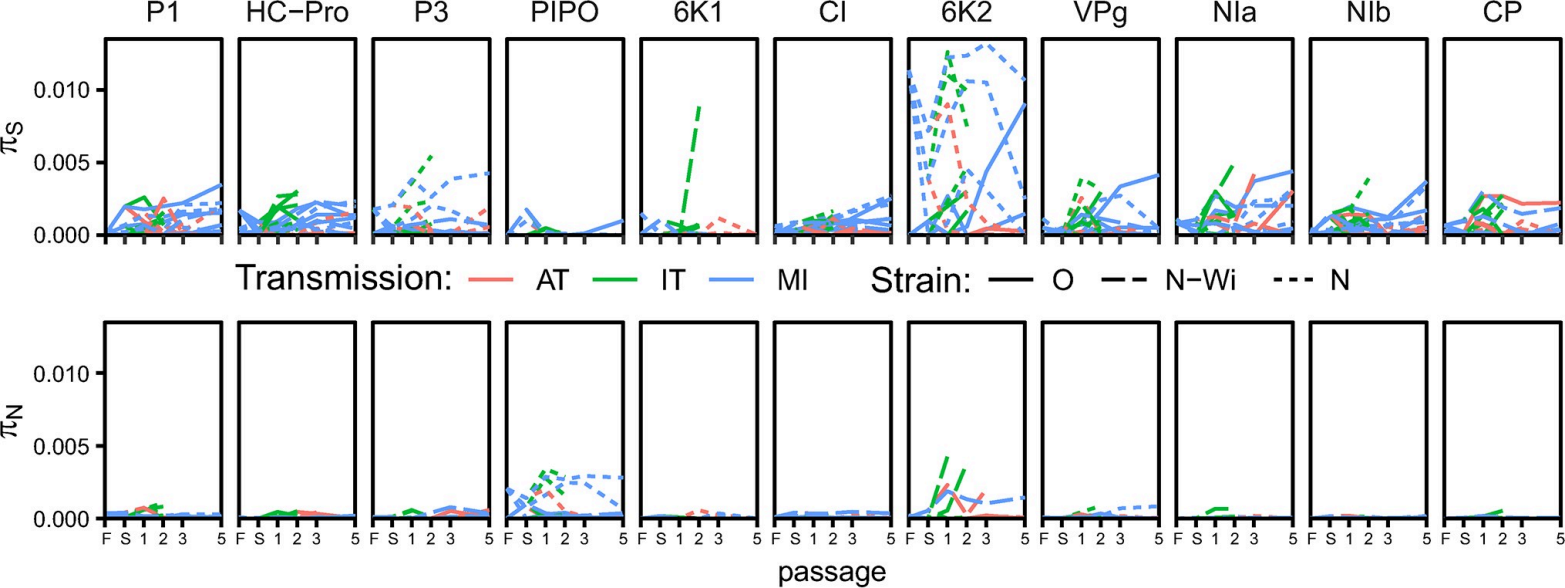
Temporal changes in nucleotide diversities for synonymous (*π*_*s*_) and nonsynonymous (*π*_*N*_) sites of different PVY cistrons (for the viral populations in leaves) during experimental evolution using three different transmission modes. Lines connect PVY nucleotide diversities for separate lineages over the course of the experiment. Colors represent three different transmission modes; line types represent three different PVY strains used in the experiment. F–Founding, S–Source.

Next, we divided the nucleotide diversity by PVY cistron and determined how the *π*_*S*_ and *π*_*N*_ for each specific cistron changed through the experiment (Fig 6). We observed larger increases in *π*_*S*_ for 6K2, compared to the other cistrons. We also observed an increase in *π*_*N*_ for 6K2, but also for P3N-PIPO. Inspection of the cumulative distribution function for all the *π*_*S*_ or *π*_*N*_ values for each of the cistrons confirmed the observed pattern (S8 Fig). However, Kolmogorov-Smirnov tests comparing all the possible pairs of cistrons' cumulative distributions for *π*_*S*_ did not show a significant differentiation of 6K2 from the other cistrons and similarly, no significant differentiation of 6K2 and P3N-PIPO was detected for *π*_*N*_ (S8 Fig). Thus, we observed indications for different selection regimes for different PVY cistrons, however, we could not confirm the differences on the basis of the data generated in this experiment.

## Discussion

We observed a significant impact of PVY transmission modes on the within-plant diversity of the PVY populations. Viral diversities were consistently low in all of the samples collected from AT lineages, as expected due to the strong population bottlenecks demonstrated for aphid transmission of PVY [8]. The observed low *π* values in the AT lineages were congruent with the estimated *N*_*e*_ values for the same lineages. The estimated *N*_*e*_ values were in the range (less than 10 to 25, for 10 aphids used for transmission) comparable to the empirically determined number of PVY viral particles transmitted by aphids (0.5 to 3.2 per single aphid) [8]. Furthermore, all the experiments were conducted using three isolates of different PVY strains, to test for possible differences in transmission for different strains. For the AT lineages, we obtained similar estimates of *N*_*e*_ values for PVY^O^ and PVY^N-Wi^ strains, however, the *N*_*e*_ values for PVY^N^ strains were higher for two of three experimental lineages. We can speculate this may imply a larger number of viral particles transmitted by aphids for this strain (either larger number of particles transmitted by a single aphid or a larger chance of virus acquisition and transfer by aphids). Differences in the efficiency of transmission of different PVY strains by aphids could have important implications in the field, where they could lead to changes in prevalence of PVY strains infecting the potato crop. However, further experiments would be needed to confirm this observation, since the transmission efficiency of different PVY strains by green peach aphid can be influenced by many different factors. Transmission efficiency for PVY^N^ and PVY^N-Wi^ was shown not to differ in a previous study [34], which is consistent with the *N*_*e*_ values estimated for this two strains in our study. Even though some studies demonstrated different efficiency of transmission for different PVY strains [35], conclusions between different studies are not consistent [36] and can be influenced by, *e*.*g*., potato cultivar, viral titer in the source leaf [37,38], virus strain, virus isolate, aphid species and clonal populations [25].

The diversities of viral populations were higher in the MI mode than in the AT mode, and they increased during the experiment. This observation agreed with the estimation of consistently higher *N*_*e*_ values for MI lineages (from 17 to 32). Nevertheless, these estimates are still relatively low, considering high number of viral particles expected to be present in an infected leaf. Previous research on contact transmission of another plant virus demonstrated that, even though the source material for mechanical transmission of viruses might contain very high number of viral particles, *e*.*g*., up to 10^9^ tobacco mosaic virus particles in a tobacco leaf [12]; only a few particles (2–20) established an infection [39], suggesting a saturation of the number of the transmitted viral units already at low numbers. It was also shown that the movement of virus from inoculated to systemic leaves induce additional bottlenecks [16], which have major contribution to the reduction in the observed *N*_*e*_, likely also in our experiment. We demonstrated in this experiment that the *N*_*e*_ for the MI mode is indeed relatively low, but still significantly higher than for the AT or IT modes. This observation has wide implications in plant virology research. The studies performed in laboratory are predominately performed using mechanical transmission, which is expected to be less frequent in the field. Quantitative estimation of the difference in a population bottlenecks expected from mechanical transmission in comparison to aphid transmission or transmission via vegetative propagation suggests that mechanical-transmission based experiments would be subjected to smaller effects of genetic drift, compared to the processes in the field.

For the IT mode, we observed increases in PVY population diversity throughout the experiment. Viral diversities for IT lineages were higher than for the AT or MI lineages. However, the estimated *N*_*e*_ values for IT lineages were very low (11 in one, and 10 or less in all the others). Even though this result seems counter intuitive, it makes sense, when inspecting the dynamics of separate SNPs in the population. Most of the variability in the IT lineages was generated *de novo* after each passage (most SNPs from previous passage were eliminated, and new SNPs appeared) (Fig 5) inferring that the number of viral genomes from the previous generation that contribute to the next generation (*N*_*e*_) was indeed very low.

Comparison of *π* for leaves and tubers of the same plants revealed significantly higher population diversities in tubers for AT and MI transmission modes and slightly lower (although, not significant) diversity in tubers for IT transmission modes (Fig 2). Observed results could be explained by considering the movement of the virus from the source infection point throughout the plant. Several studies have demonstrated that viral systemic movement within the plants (between different parts of the plant, *e*.*g*., from leaf to leaf) is accompanied by significant genetic bottlenecks [40–43], which might be influenced by different source-to-sink movements of virions via the plant phloem [16,42].

**Fig 7:**
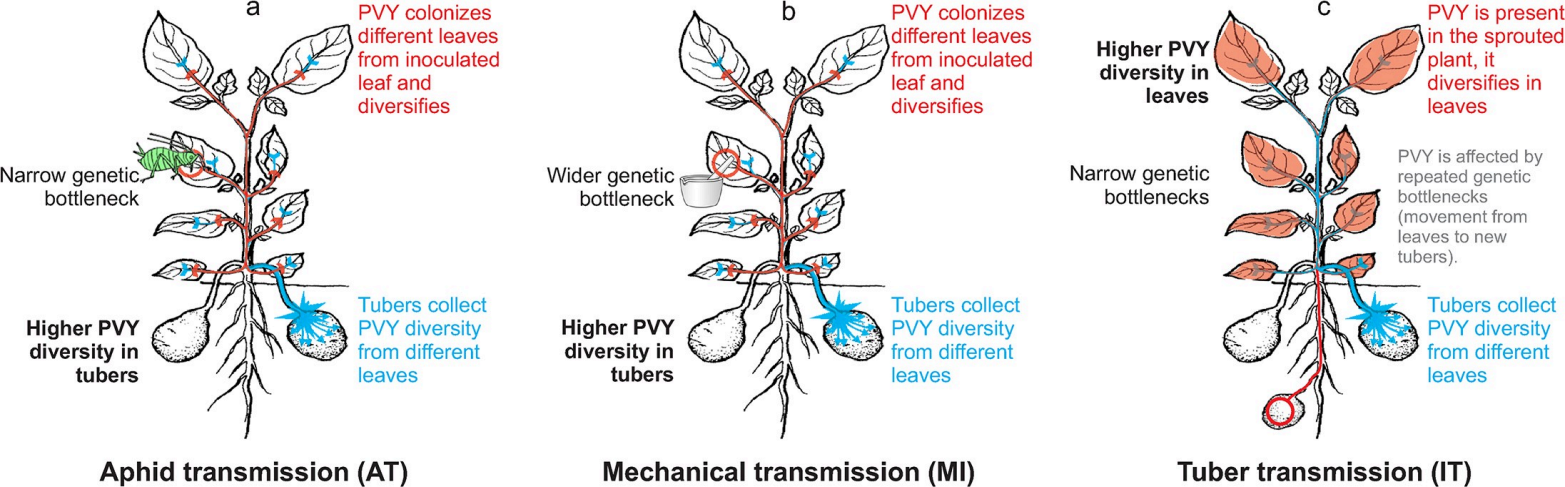
Summary of observed patterns in the PVY populations’ dynamics for different transmission modes. In black, on the left side of potato plant drawings are the representations of the population dynamics' patterns. Suggested mechanisms explaining the observed patterns are highlighted in red, blue, and grey on potato plant drawings and on their right side. (a) Aphid transmission mode, (b) mechanical transmission mode, and (c) tuber transmission mode.

In our experiments, in the AT and MI modes, virus enters a plant through the inoculated leaf and then moves to other leaves (Fig 7A and 7B) via source-to-sink transport in the phloem. These movements impose repeated population bottlenecks for the virus that can re-diversify in each of the leaves over time. As tubers develop they act as a sink and virus moves to the tubers via the phloem from different leaves (source). Population bottlenecks likely occur during the movement of virus from leaves to the tubers. Nevertheless, for the AT and MI modes, the observed virus populations within tubers are more diverse than the populations within a single leaf (Fig 7A and 7B), which can be most likely attributed to a high level of admixture of viral variants in tubers (*i*.*e*., as a sink, tubers collect variants from many leaves).

On the other hand, in the IT mode, the virus is present already in the sprouted plantlet and thus has more time to diversify (more replication cycles) in each of the leaves, compared to the MI and AT modes. This might contribute to higher observed diversity of the viral populations in leaves in the IT mode, compared to the MI and AT modes (Fig 2). In the IT mode, we observed similar or slightly lower diversities of the viral populations in tubers than in leaves (Fig 7C). The observed ratio might be the consequence of combined effect of viral-movement induced bottlenecks, population admixture in tubers and higher number of virus replication cycles in leaves for the IT mode.

The differences in population diversities between leaves and tubers, and the observation that *N*_*e*_ do not always correlate with observed *π*, highlight the fact that transmission event itself cannot be the only factor explaining the underlying differences in population structures of PVY within plants in different transmission modes. Differences in the number of replication cycles of the virus, movement of the virus within the plant, admixture etc., need to be considered to better understand how the structure of within-plant virus population is shaped by different transmission modes.

Although we detected several SNPs rising in frequency or even being fixed in the populations (especially in AT mode), it was not possible to distinguish, due to the small estimated *N*_*e*_ values, if these changes could be attributed to selection and/or genetic drift. It is possible that both, strong genetic drift and selection, contribute to the observed patterns; however, different experimental approaches would be needed to test such hypotheses. When investigating the position of fixed nonsynonymous mutations, we did not observe SNPs causing changes directly in the motifs known to be involved in the PVY-aphid interactions in any lineage. Nevertheless, we observed a fixed change in lineage AT3 that might be particularly interesting; it is located in HC-Pro (conservative substitution D334E), in the relatively close proximity (15 amino acids upstream) to the PTK motif, which is known to interact with CP during the aphid transmission. Moreover, observed increases in nucleotide diversities for the 6K2 and P3N-PIPO cistrons hint that these may be under different selection pressures than other PVY cistrons.

The observed patterns of population structure changes for different transmission modes confirmed theoretical expectations and revealed some new findings about the processes underlying the transmission of PVY. The results are especially interesting from the perspective of the ecosystem. The observed low diversities and strong genetic bottlenecks for the within-plant virus populations in AT mode were also accompanied by frequent fixation of minor or new alleles in different lineages (Fig 5). Thus, even though the within-plant diversities were low, fixation of new or minor alleles in the populations could finally generate a higher diversity among all of the plants in the field. This is supported also by observing how divergence of the viral populations changes from the starting reference sequence (S9 Fig)–even though the within-plant diversities (Fig 2) in the MI lineages increased and were higher than those in the AT lineages, proxies for divergence (*d*_*S*_ and *d*_*N*_) for lineages of both transmission modes are very similar (S9 Fig). One might speculate that if similar trends would continue for more passages, the divergence of the AT lineages would likely surpass those of the MI lineages (since more fixed mutations would accumulate).

Transmission of potyviruses by aphids have an important epidemiological role for the spread of the virus mostly on relatively short distances [44], *e*.*g*., within a field. Here, according to our results, frequent and abrupt declines in within-plant-virus population diversities, accompanied by the fixation of new variants, will cause the divergence between the viral populations in different plants and effectively translocate them on a different position in the adaptation landscape. On the other hand, the mechanical transmission (or transmission by wounding), which is the main experimental approach for virus transmission, is less likely to occur in the field [25]. It will enable transmission of larger population sizes, lessening the effect of the genetic drift and, as observed, causing the viral populations to retain diversity, being centered around the similar position in a sequence space. Tuber transmission, which is important in transmission of virus from year to year and over long distances [25], will in contrast to AT generate a higher diversity of viral populations locally evolving around different peaks in the adaptation landscape; however, the genetic admixture in tubers would prevent an efficient fixation of new alleles. These observations have implications for the understating of the evolution of PVY, an important plant pathogen, in the field. It was suggested that reducing the *N*_*e*_ (inducing stronger bottlenecks, such as in AT) of PVY populations in plants might slow down virus adaptation and consequently, the emergence of resistant-breaking virus isolates [45]. However, one needs to consider that different transmission modes and other processes in viral life cycle influence the *N*_*e*_ of the populations simultaneously and subsequently. Based on our results, we can hypothesize that tuber transmission represents a potent generator of diverse viral populations, providing new alleles to the PVY metapopulation (field-wise) and the diversity can then be (randomly) subsampled, when PVY is spreading in field through aphid transmission. We cannot directly link these processes to predict, *e*.*g*., the severity of disease in the field, however, the quantitative data presented in this paper enable the extension of epidemiology models of plant virus diseases by integrating the population genetics parameters underlying different transmission modes. For example, under the scenarios described above, we can predict reduced within-plant and increased within-field diversity for PVY, when aphid transmission is not decreased by control measures. At the same time, one could expect increased diversity of PVY populations within infected plants generated through vegetative propagation (tubers) or via mechanical transmission. Carefully planned experimental work and/or *in-silico* simulations would be needed to determine how the described differences in population genetic parameters for different transmission modes influence the adaptation of the virus to different environmental conditions (*e*.*g*., new host plant or resistant plant genotypes).

## Supporting information

S1 Table List of primer sequences used for amplification of different PVY strains. (XLSX)

S2 Table Detailed information about analyzed samples. (XLSX)

S3 Table Results of the statistical tests for *π* data for PVY populations in leaves. Results of *post-hoc* pairwise comparisons of estimated marginal means for groups defined by factors *STRAIN*, *TRANSMISSION MODE*, *PASSAGE* and *TRANSMISSION MODE*×*PASSAGE*.
(XLSX)

S1 Fig Mapped read depths across the PVY^O^ genome. Each line represents mapped read depth across the genome of the virus for each of the PVY^O^ samples in the experiment. Samples are color coded according to the legend below the plot. The mapped read depth values are log_10_-transformed.
(PDF)

S2 Fig Mapped read depths across the PVY^N^ genome. Each line represents mapped read depth across the genome of the virus for each of the PVY^N^ samples in the experiment. Samples are color coded according to the legend below the plot. The mapped read depth values are log_10_-transformed.
(PDF)

S3 Fig Mapped read depths across the PVY^N-Wi^ genome. Each line represents mapped read depth across the genome of the virus for each of the PVY^N-Wi^ samples in the experiment. Samples are color coded according to the legend below the plot. The mapped read depth values are log_10_-transformed.
(PDF)

S4 Fig Locus-specific estimations of selection coefficients (*s*) for lineages of PVY^O^. Each plot represents results of the s values estimations for a separate evolutionary lineage of the experiment. Horizontal lines represent 95% highest posterior density intervals for SNPs *s* value estimations, blue dots represent their median and green dots their mean. AT–aphid transmission, MI–mechanical transmission, IT–tuber transmission.
(PDF)

S5 Fig Locus-specific estimations of selection coefficients (*s*) for lineages of PVY^N^. Each plot represents results of the *s* values estimations for a separate evolutionary lineage of the experiment. Horizontal lines represent 95% highest posterior density intervals for SNPs *s* value estimations, blue dots represent their median and green dots their mean. AT–aphid transmission, MI–mechanical transmission, IT–tuber transmission.
(PDF)

S6 Fig Locus-specific estimations of selection coefficients (*s*) for lineages of PVY^N-Wi^. Each plot represents results of the *s* values estimations for a separate evolutionary lineage of the experiment. Horizontal lines represent 95% highest posterior density intervals for SNPs *s* value estimations, blue dots represent their median and green dots their mean. AT–aphid transmission, MI–mechanical transmission, IT–tuber transmission.
(PDF)

S7 Fig Dynamics of SNPs that reached at least 10% in more than one lineage. The heatmap within each rectangle represent dynamics of SNPs frequencies in particular lineage during the experiment. Lines represent specific SNPs, rows represent passages (S–source); SNPs frequencies are represented by color according to the color key at the bottom of the figure.
(PDF)

S8 Fig Cumulative distribution functions and pairwise Kolmogorov-Smirnov tests for *π* data for separate PVY cistrons. Upper panel represents cumulative distribution functions for *π*_*S*_ data for all the samples from the experiment separated by cistron (color coded). Lower panel represents cumulative distribution functions for *π*_*N*_ data for all the samples from the experiment separated by cistron (color coded). Within the panels the networks summarize the results of Kolmogorov-Smirnov tests for the same data (edges connect the cistron pairs for which Kolmogorov-Smirnov test did not show a significant difference).
(PDF)

S9 Fig Temporal changes in *d*_*S*_ and *d*_*N*_ for PVY populations in leaves during experimental evolution using three different transmission modes. Thin lines connect *d*_*S*_ (upper panel) or *d*_*N*_ (lower panel) values for separate lineages over the course of the experiment. Colors represent three different transmission modes (AT–aphid transmission, IT–transmission through infected tubers and MI–mechanical transmission), line types represent three different PVY strains used in the experiment. Thick translucent lines connect average values for all of the lineages within the same transmission mode over the course of the experiment.
(PDF)

S1 File Filtered SNP tables for all of the samples analyzed in the study. (ZIP)
