# Supplementary material for: Transmission modes affect the population structure of potato virus Y in potato
Source: PLoS Pathog. 2020 Jun 23;16(6):e1008608. doi: 10.1371/journal.ppat.1008608 (PMC7347233; doi:10.1371/journal.ppat.1008608)

S2 Figure. Mapped read depths across the PVY^N genome. Each line represents mapped read depth across the genome of the virus for each of the PVY^N samples in the experiment. Samples are color coded according to the legend below the plot. The mapped read depth values are log_{10} transformed.

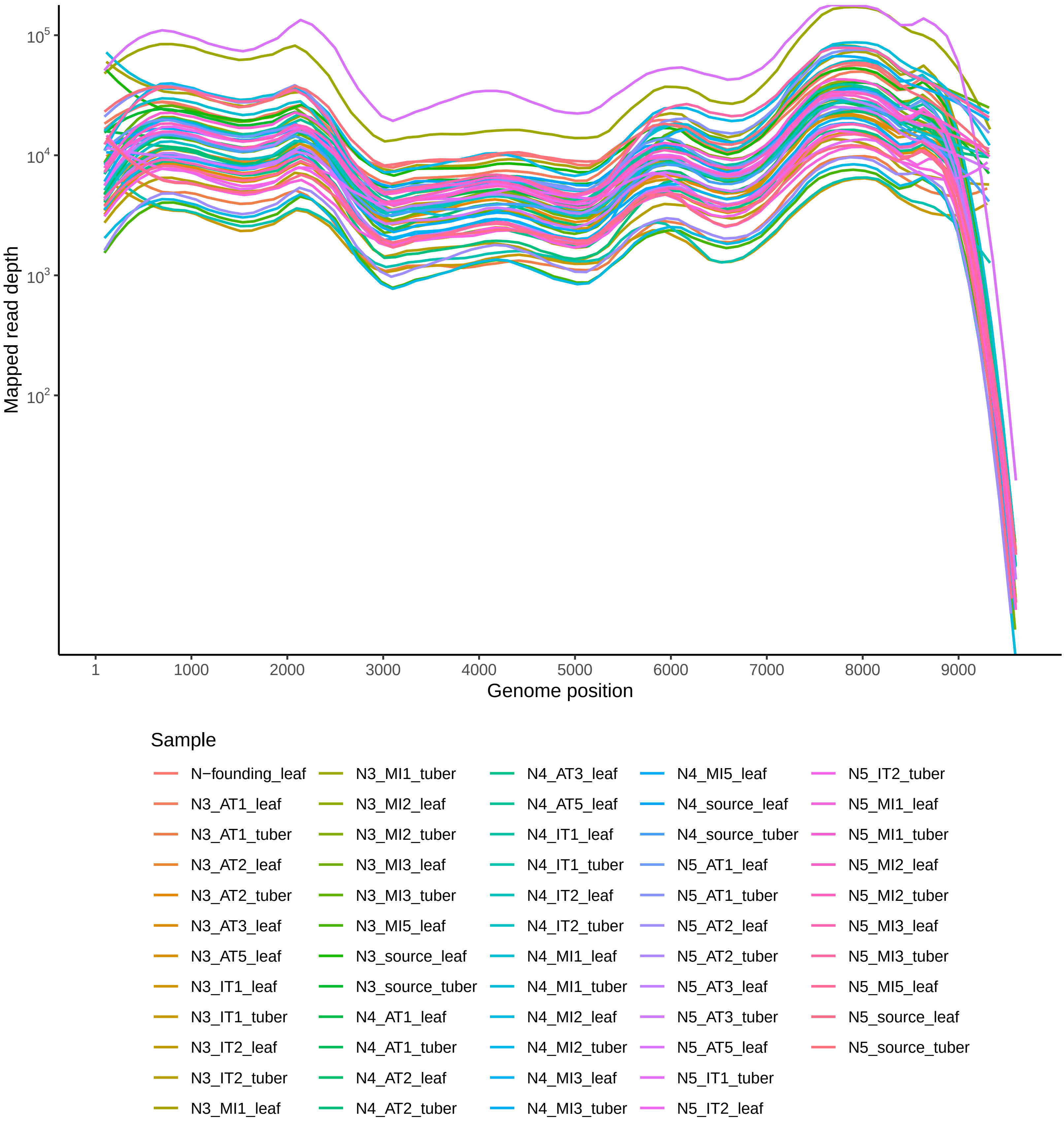

Supplement: S2 Fig — Each line represents mapped read depth across the genome of the virus for each of the PVYN samples in the experiment. Samples are color coded according to the legend below the plot. The mapped read depth values are log10-transformed. (PDF) [file ppat.1008608.s005.pdf]
