# Supplementary material for: Transmission modes affect the population structure of potato virus Y in potato
Source: PLoS Pathog. 2020 Jun 23;16(6):e1008608. doi: 10.1371/journal.ppat.1008608 (PMC7347233; doi:10.1371/journal.ppat.1008608)

S3 Figure. Mapped read depths across the PVY$^{N\text{-}Wi}$ genome. Each line represents mapped read depth across the genome of the virus for each of the PVY$^{N\text{-}Wi}$ samples in the experiment. Samples are color coded according to the legend below the plot. The mapped read depth values are $\log_{10}$ transformed.

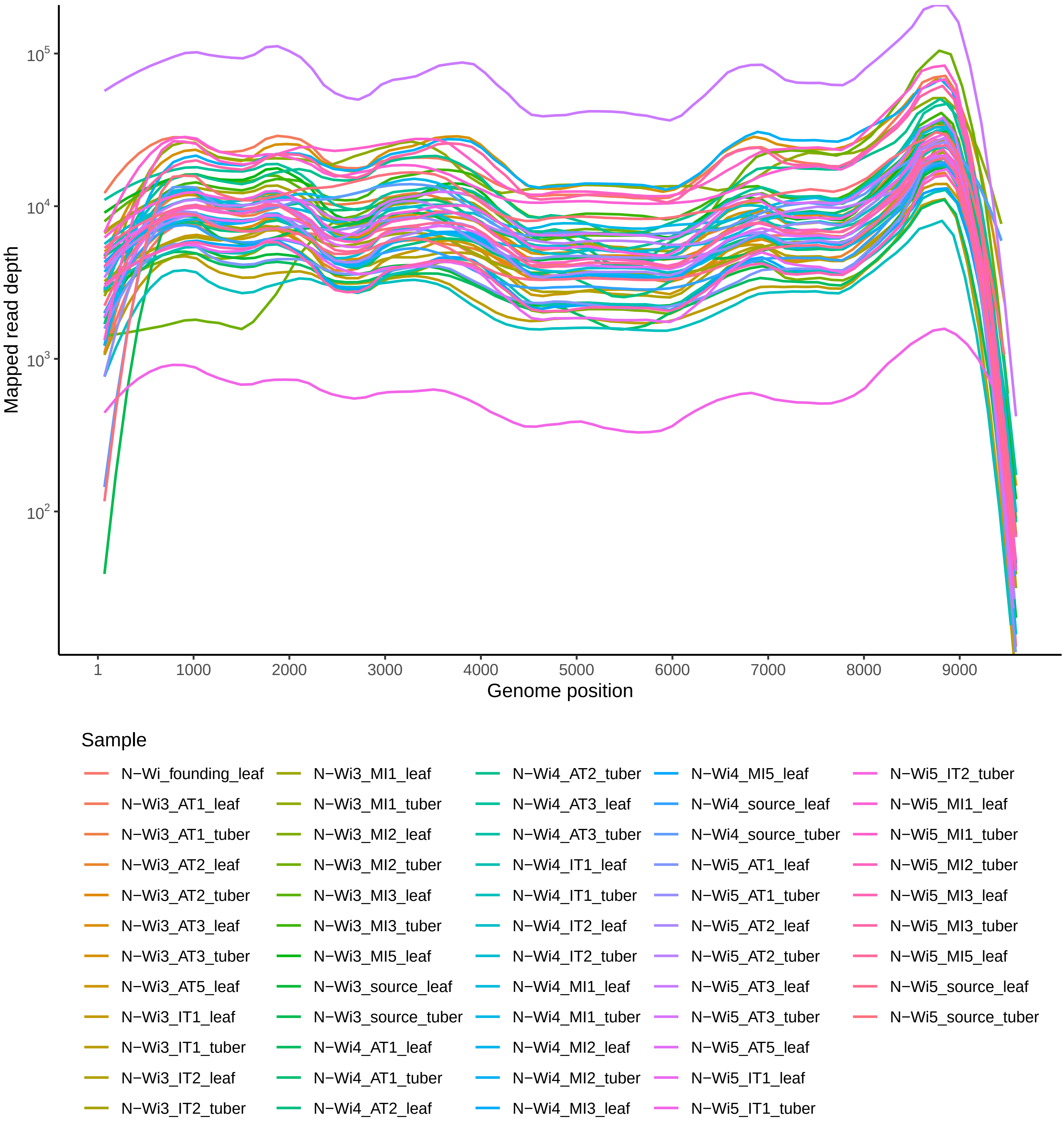

Supplement: S3 Fig — Each line represents mapped read depth across the genome of the virus for each of the PVYN-Wi samples in the experiment. Samples are color coded according to the legend below the plot. The mapped read depth values are log10-transformed. (PDF) [file ppat.1008608.s006.pdf]
