# Supplementary figures and images for: Transmission modes affect the population structure of potato virus Y in potato
Source: PLoS Pathog. 2020 Jun 23;16(6):e1008608. doi: 10.1371/journal.ppat.1008608 (PMC7347233; doi:10.1371/journal.ppat.1008608)

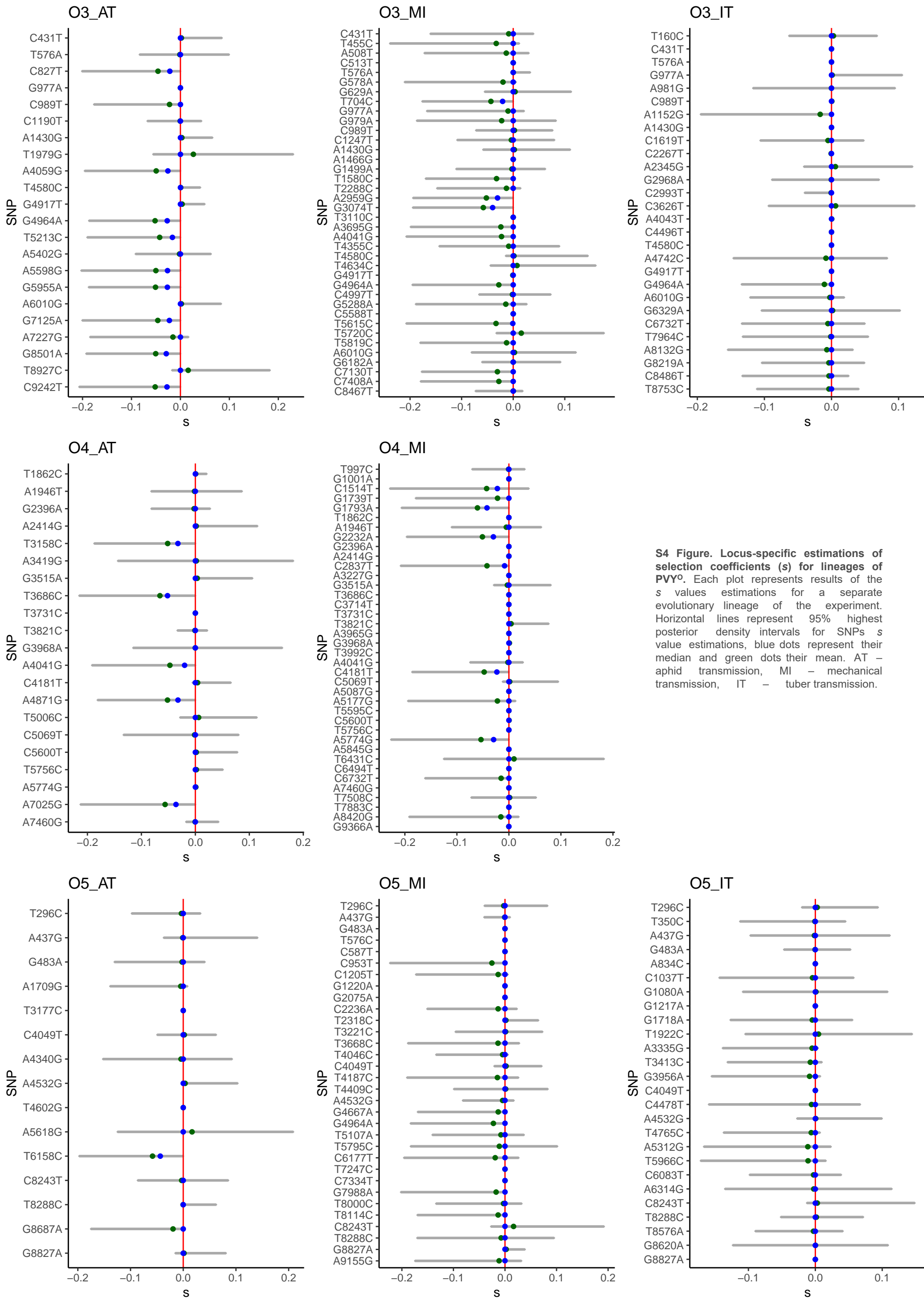

Supplement: S4 Fig — Each plot represents results of the s values estimations for a separate evolutionary lineage of the experiment. Horizontal lines represent 95% highest posterior density intervals for SNPs s value estimations, blue dots represent their median and green dots their mean. AT–aphid transmission, MI–mechanical transmission, IT–tuber transmission. (PDF) [file ppat.1008608.s007.pdf]

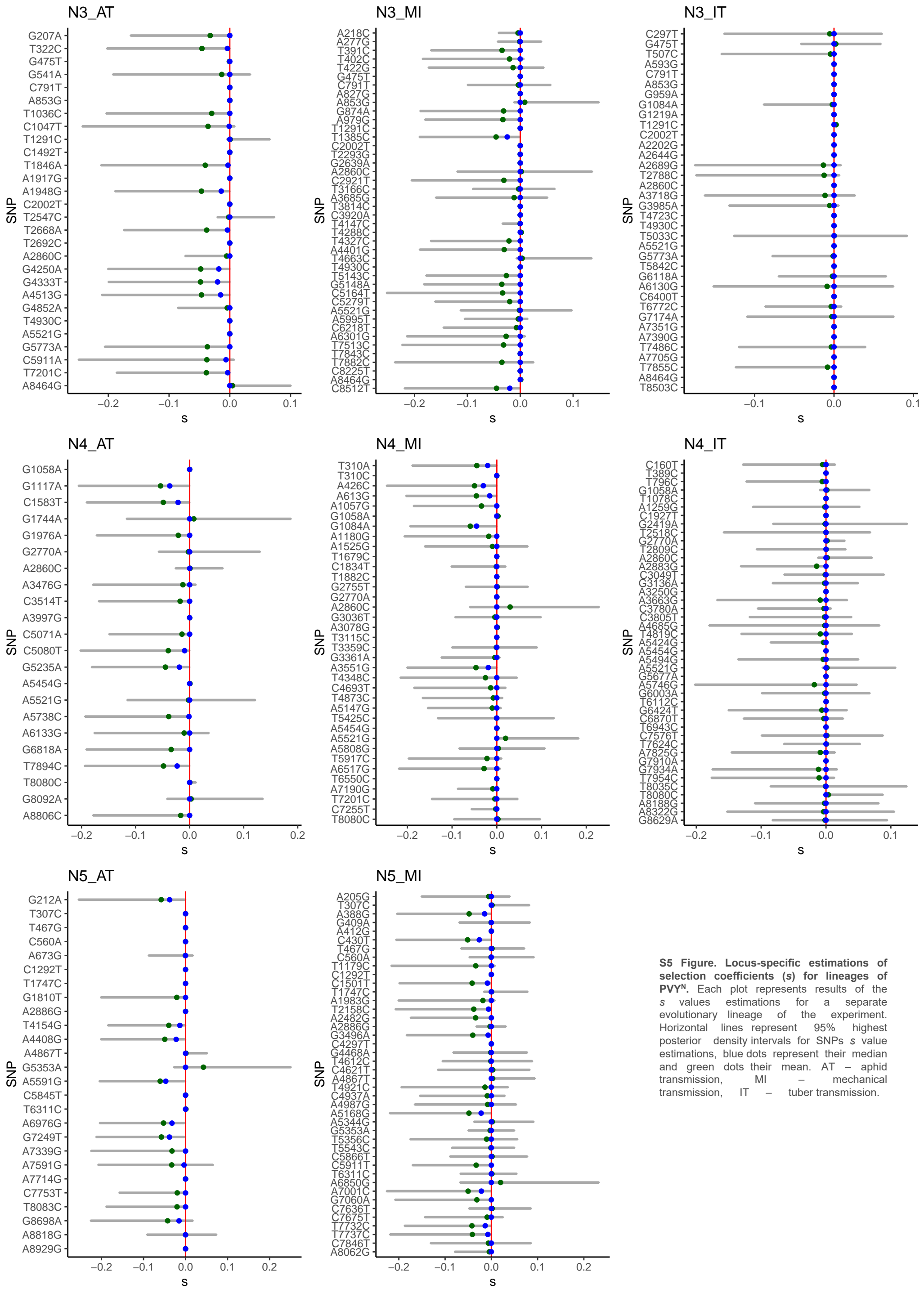

Supplement: S5 Fig — Each plot represents results of the s values estimations for a separate evolutionary lineage of the experiment. Horizontal lines represent 95% highest posterior density intervals for SNPs s value estimations, blue dots represent their median and green dots their mean. AT–aphid transmission, MI–mechanical transmission, IT–tuber transmission. (PDF) [file ppat.1008608.s008.pdf]

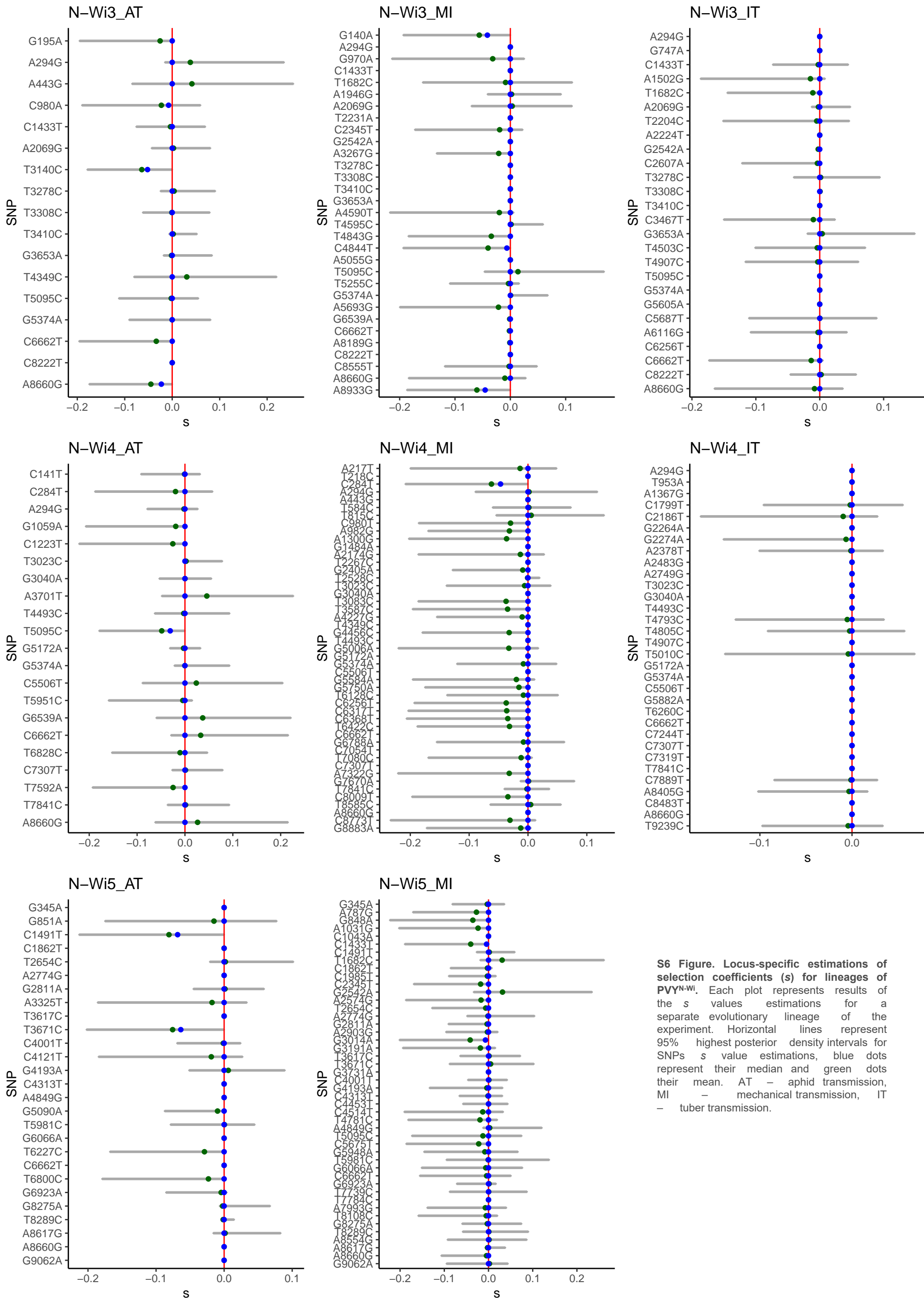

Supplement: S6 Fig — Each plot represents results of the s values estimations for a separate evolutionary lineage of the experiment. Horizontal lines represent 95% highest posterior density intervals for SNPs s value estimations, blue dots represent their median and green dots their mean. AT–aphid transmission, MI–mechanical transmission, IT–tuber transmission. (PDF) [file ppat.1008608.s009.pdf]
