# Supplementary material for: Transmission modes affect the population structure of potato virus Y in potato
Source: PLoS Pathog. 2020 Jun 23;16(6):e1008608. doi: 10.1371/journal.ppat.1008608 (PMC7347233; doi:10.1371/journal.ppat.1008608)
Supplement: S8 Fig — Upper panel represents cumulative distribution functions for πS data for all the samples from the experiment separated by cistron (color coded). Lower panel represents cumulative distribution functions for πN data for all the samples from the experiment separated by cistron (color coded). Within the panels the networks summarize the results of Kolmogorov-Smirnov tests for the same data (edges connect the cistron pairs for which Kolmogorov-Smirnov test did not show a significant difference). (PDF) [file ppat.1008608.s011.pdf]

S8 Figure. Cumulative distribution functions and pairwise Kolmogorov-Smirnov tests for π data for separate PVY cistrons. Upper panel represents cumulative distribution functions for π_S data for all the samples from the experiment separated by cistron (color coded). Lower panel represents cumulative distribution functions for π_N data for all the samples from the experiment separated by cistron (color coded). Within the panels the networks summarize the results of Kolmogorov-Smirnov tests for the same data (edges connect the cistron pairs for which Kolmogorov-Smirnov test did not show a significant difference).

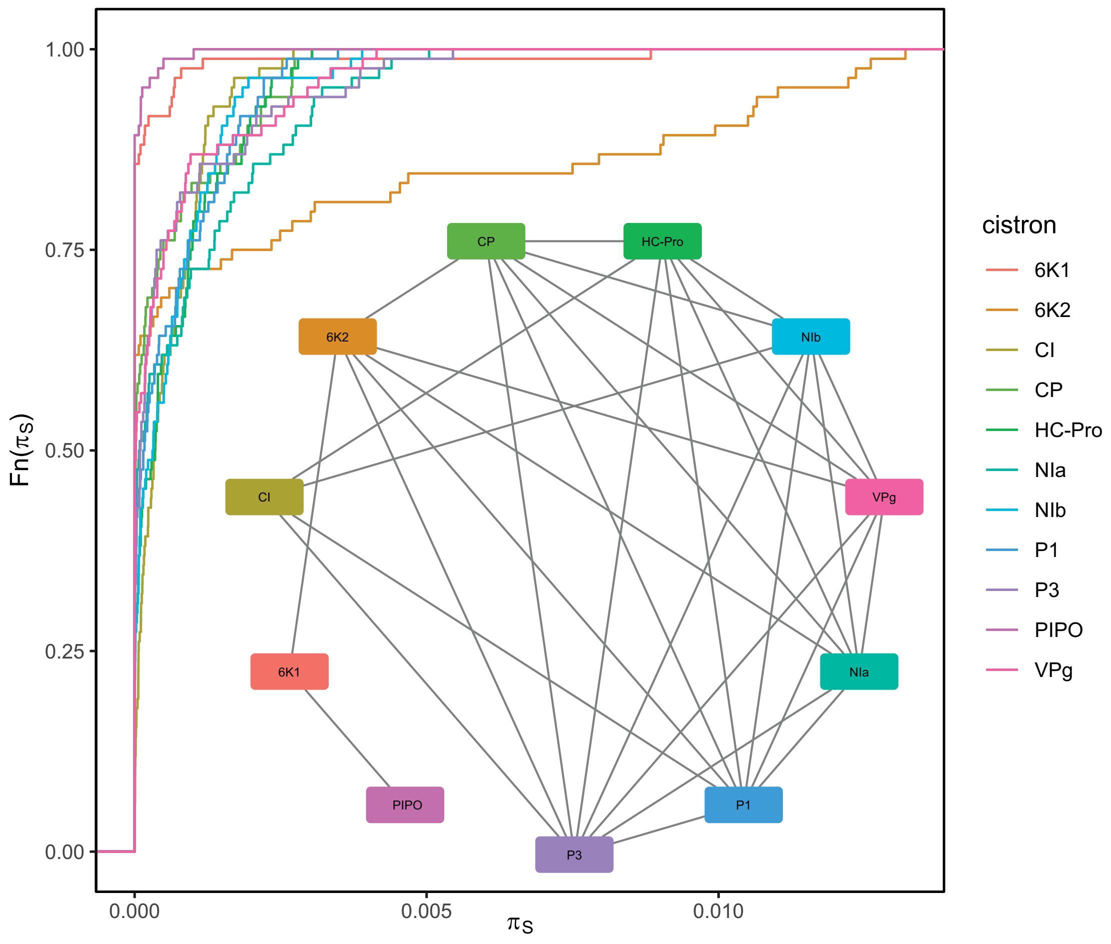

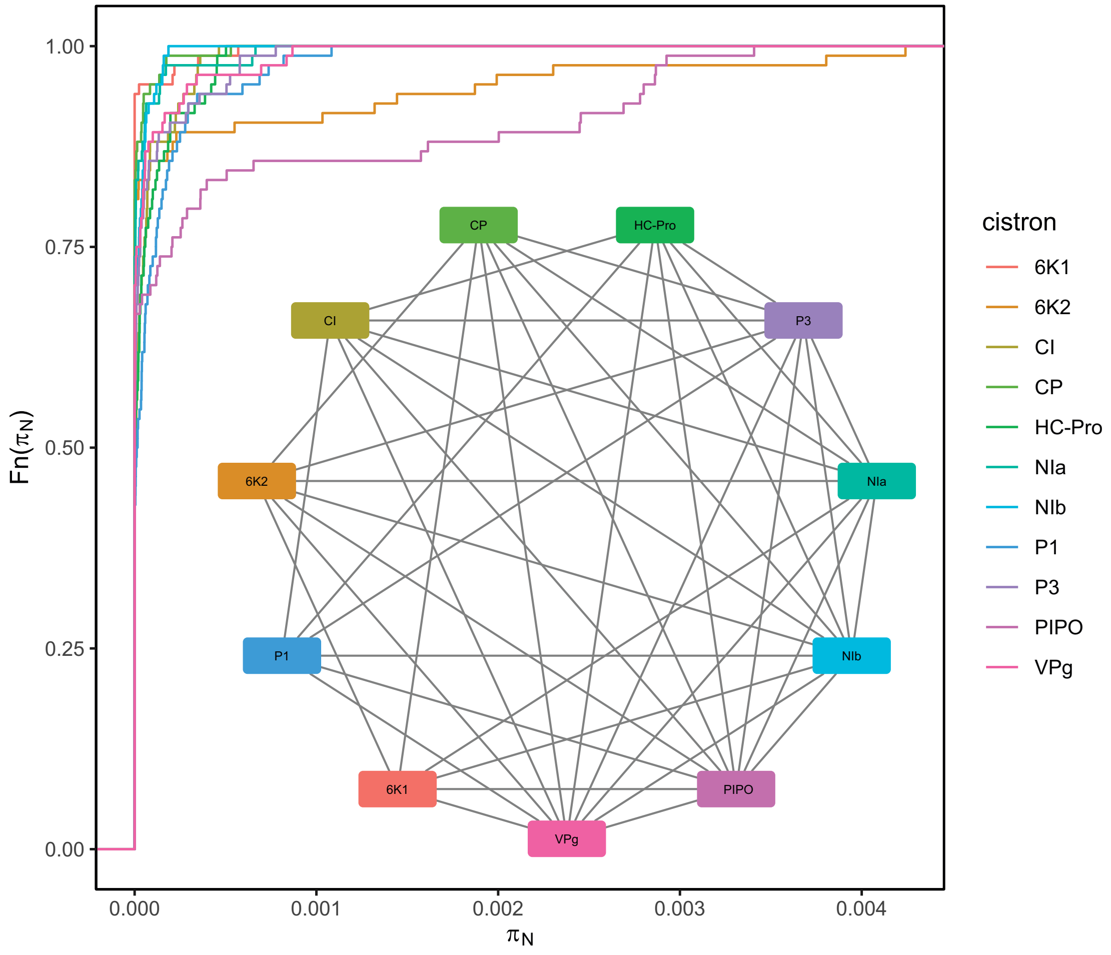

Comparison of cumulative distribution functions for different cistrons (color-coded lines) informs us about how similar/different the distributions of the analyzed values (π_S or π_N) are for different cistrons - similar shapes and positions of the functions imply similar distribution of the values.

For example, on the upper panel (π_S) we can recognize three „groups" of cistrons: 6K1(red) and PIPO (bright pink) in the first group, most of the cistrons in the second group and 6K2 (orange) in the third group.

Kolmogorov-Smirnov tests represent a formal way to test for a statistically significant differences in location and shape of the pairs of cumulative distributions. The network connects the pairs of the cistrons for which the test did not show a significant difference. Thus, the cistrons that are less connected are the ones, for which the distribution of analyzed values was most different from the distributions of the rest of the citrons (e.g., PIPO and 6K1 for π_S).
