# Supplementary material for: Transmission modes affect the population structure of potato virus Y in potato
Source: PLoS Pathog. 2020 Jun 23;16(6):e1008608. doi: 10.1371/journal.ppat.1008608 (PMC7347233; doi:10.1371/journal.ppat.1008608)

S9 Figure. Temporal changes in d_S and d_N for PVY populations in leaves during experimental evolution using three different transmission modes. Thin lines connect d_S (upper panel) or d_N (lower panel) values for separate lineages over the course of the experiment. Colors represent three different transmission modes (AT – aphid transmission, IT – transmission through infected tubers and MI – mechanical transmission), line types represent three different PVY strains used in the experiment. Thick translucent lines connect average values for all of the lineages within the same transmission mode over the course of the experiment.

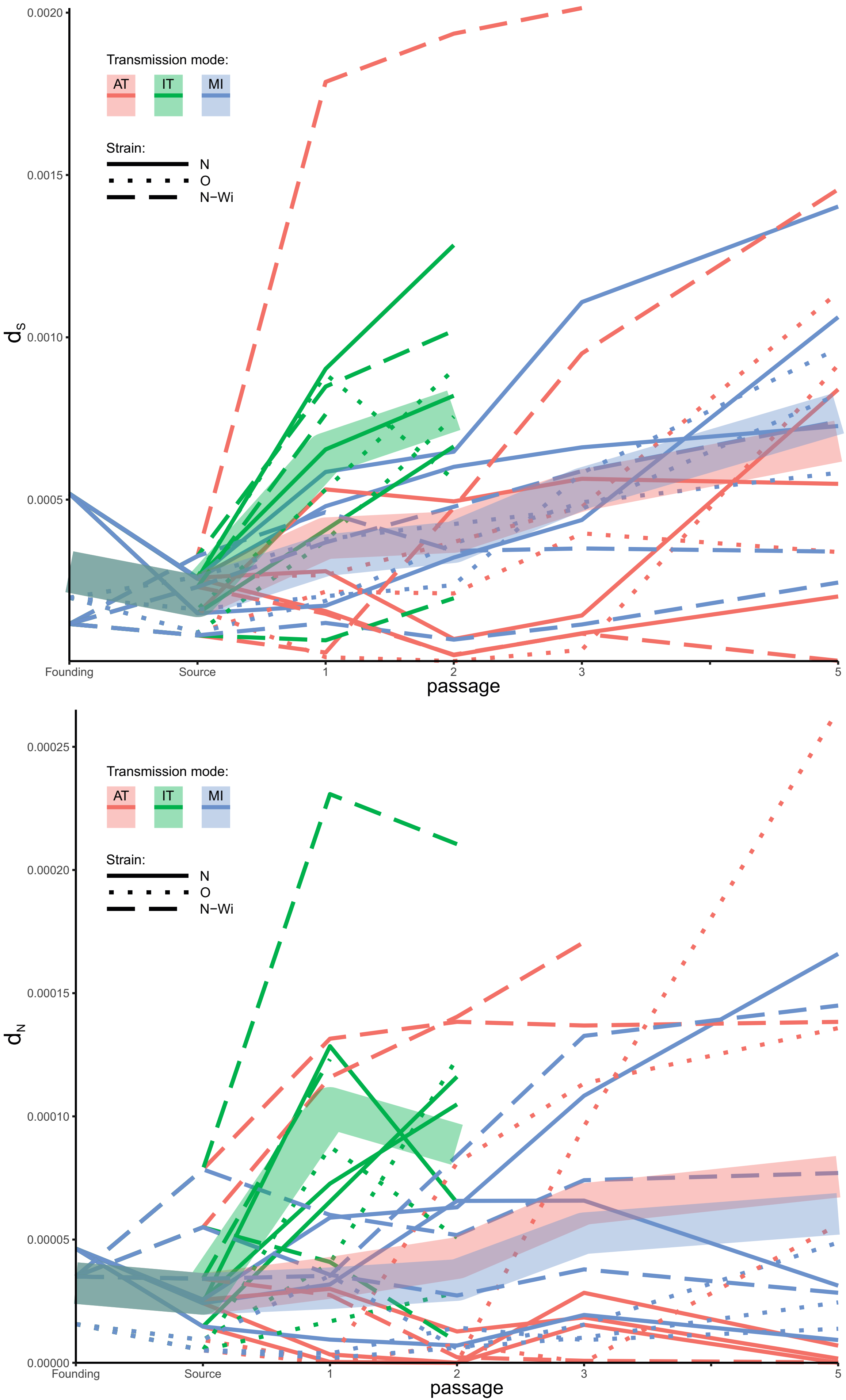

Supplement: S9 Fig — Thin lines connect dS (upper panel) or dN (lower panel) values for separate lineages over the course of the experiment. Colors represent three different transmission modes (AT–aphid transmission, IT–transmission through infected tubers and MI–mechanical transmission), line types represent three different PVY strains used in the experiment. Thick translucent lines connect average values for all of the lineages within the same transmission mode over the course of the experiment. (PDF) [file ppat.1008608.s012.pdf]
