# Supplementary material for: Transmission modes affect the population structure of potato virus Y in potato
Source: PLoS Pathog. 2020 Jun 23;16(6):e1008608. doi: 10.1371/journal.ppat.1008608 (PMC7347233; doi:10.1371/journal.ppat.1008608)

S7 Figure. Dynamics of SNPs that reached at least 10% in more than one lineage. The heatmap within each rectangle represent dynamics of SNPs frequencies in particular lineage during the experiment. Lines represent specific SNPs, rows represent passages (S – source); SNPs frequencies are represented by color according to the color key at the bottom of the figure.

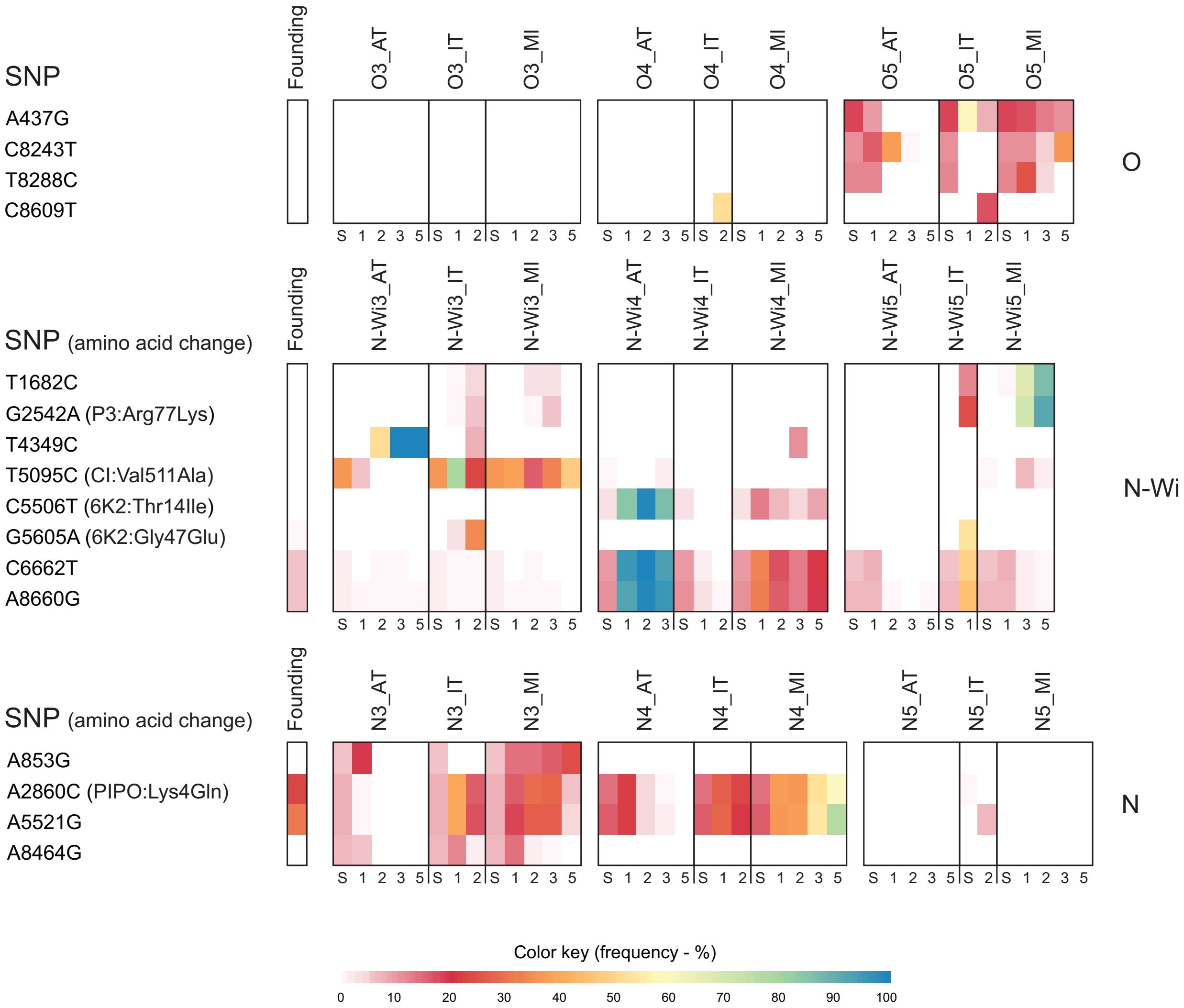

Supplement: S7 Fig — The heatmap within each rectangle represent dynamics of SNPs frequencies in particular lineage during the experiment. Lines represent specific SNPs, rows represent passages (S–source); SNPs frequencies are represented by color according to the color key at the bottom of the figure. (PDF) [file ppat.1008608.s010.pdf]
